# Spectroscopic Study of a Novel Binaphthyl Amine Fluorescent Probe for Chiral Recognition of D/L-Lysine

**DOI:** 10.3390/ijms25147504

**Published:** 2024-07-09

**Authors:** Liji Wu, Xiangyu Lu, Wentao Cai, Yajun Zou, Xiaoyu Zhang, Jialiang Yang, Gang Zhao

**Affiliations:** School of Chemical Engineering, Sichuan University, Chengdu 610207, China; 2020223075216@stu.scu.edu.cn (L.W.); 2022323070047@stu.scu.edu.cn (X.L.); 2019223070112@stu.scu.edu.cn (W.C.); 2021223075220@stu.scu.edu.cn (Y.Z.); 2022223070114@stu.scu.edu.cn (X.Z.); 2021323070043@stu.scu.edu.cn (J.Y.)

**Keywords:** chiral lysine recognition, BINAM-based fluorescent probes, enantioselectivity, fluorescence analysis

## Abstract

Lysine plays a crucial role in promoting development, enhancing immune function, and improving the function of central nervous system tissues. The two configurational isomers of amino acids have significantly different effects. Currently, methods for chiral recognition of lysine have been reported; however, previous detection methods have drawbacks such as expensive equipment and complicated detection processes. Fluorescence analysis, on the other hand, boasts high sensitivity, strong selectivity, and simple operation. In this study, we synthesized four novel Binaphthyl-Amine (BINAM)-based fluorescent probes capable of specifically identifying the L-configuration of lysine among the twenty amino acids that constitute human proteins. The enantiomeric fluorescence enhancement ratio (ef or ΔI_L_/ΔI_D_) reached up to 15.29, demonstrating high enantioselectivity. In addition, we assessed the probe’s recognition capabilities under varying pH levels, reaction times, and metal ion conditions, along with its limit of detection (LOD) and quantum yield. Our results suggest that this probe serves as a highly stable tool for the detection of chiral lysine.

## 1. Introduction

Throughout the evolutionary process, organisms gradually developed a preference for specific chiral amino acids, which enhanced their chances of survival and reproduction [1]. The existence of chiral amino acids is a result of the interplay between Earth’s early environment and biological evolution, laying crucial groundwork for the formation and development of life systems [2,3]. Amino acids are basic units that constitute the macromolecule proteins in living organisms. They are also crucial components in life processes [4]. In natural amino acids, apart from glycine, all other amino acids exist in two stereoisomeric forms, L and D. The specific spatial arrangement of L-amino acids is essential for proper protein folding, maintaining structural integrity that is critical for biological activity [5]. Additionally, the role of amino acid chirality extends to drug design, where the preferential use of L-configured amino acids enhances the biological compatibility and efficacy of pharmaceuticals [6]. The role of D-amino acids in living organisms is relatively limited, primarily demonstrated in specific areas such as antimicrobial activity, drug design, research tools, and applications in veterinary medicine and animal feed, thereby showcasing their distinct biological functions and potential utility [7]. Overall, the chiral nature of amino acids profoundly influences the structure, function, and regulation of biological systems, reflecting the evolutionary adaptability and survival advantages conferred by specific stereoisomers.

Lysine is a basic amino acid, and most higher animals cannot synthesize lysine [8]. Therefore, lysine is an essential amino acid that must be ingested in sufficient quantities from the diet to maintain protein synthesis [9]. L-lysine is considered an essential amino acid of great importance to human health, playing a significant role in enhancing immunity, promoting calcium absorption, and improving central nervous system function [10]. Abnormal metabolism of L-lysine may lead to certain cancers [11]. L-lysine has multiple functions, including enhancing immunity, promoting skeletal muscle growth, facilitating fat metabolism, and alleviating anxiety [12,13,14,15]. Additionally, it can also produce synergistic effects with other nutrients and promote their absorption, improving the utilization efficiency of various nutrients and thereby better expressing the physiological functions of different nutrients [16,17]. A deficiency of D-lysine can lead to negative health effects, such as uremia. Differences exist in the way the body absorbs and utilizes D-lysine and L-lysine, with D-lysine being absorbed and utilized less efficiently. In contrast, L-lysine provides the primary biological activity [18]. Lysine is essential for protein function. Given its broad and reliable use as a drug target, lysine has garnered significant attention [19]. Consequently, it also serves an important role in distinguishing between different enantiomers.

Although there are already some methods for detecting lysines, such as the luminol chemiluminescence method [20], gas chromatography–mass spectrometry [21], synchrotron X-ray powder diffraction, and thermogravimetric analysis [22], these methods come with the disadvantages of expensive equipment and cumbersome detection processes. Therefore, there is a need for a simple and efficient method for detecting lysine. Fluorescence analysis, with its advantages of high sensitivity, strong selectivity, low sample requirement, and simplicity, has attracted great interest from researchers. However, while it offers these advantages, the current synthesis of many fluorescent probes is challenging, and they only exhibit limited enantioselectivity. Therefore, preparing efficient fluorescent probes for the chiral recognition of lysine via a simple synthetic route remains a significant challenge.

**Figure 1:**
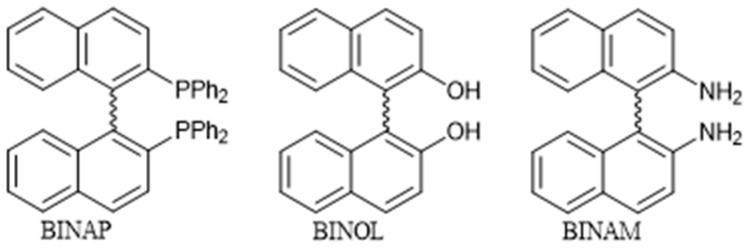
BINAP, BINOL, and BINAM.

Chiral sensors can differentiate between stereoisomers by producing distinct optical signals in their ultraviolet (UV) and fluorescence spectra. During this process, chiral sensors play a crucial role. The essence of designing chiral fluorescent sensors hinges on attaining a highly discriminative response towards two enantiomers, manifested as enantioselective spectral responses. This response is rooted in the varying affinities of the sensor’s structural framework for distinct enantiomeric substrates, establishing the microscopic foundation of chiral recognition [23]. Large chiral barriers and rigid structures are considered two useful elements in constructing chiral sensors; due to this, chiral 1,1′-binaphthyl compounds have become very commonly used chiral fluorescent groups for enantioselective recognition. Axially chiral 1.1′-Binaphthyl-2.2′-diphemyl phosphine (BINAP), 1,1′-binaphthyl-2,2′-diol (BINOL) (Figure 1), and their derivatives have become some of the most successful chiral ligands/catalysts in asymmetric catalysis and are widely used in various enantioselective catalysis processes [24]. For example, Pu outlined chiral sensors based on 1,1′-binaphthalene [25] and the efforts his group have made in developing chiral sensors based on BINOL [26]. They have also developed a variety of binaphthyl fluorescent probes with high enantioselectivity [23,27]. However, 2,2′-Diamino-1,1′-binaphthalene (BINAM), which also possesses axial chirality, has seen very limited application in asymmetric synthesis, with relatively few related publications. BINAM has long been considered by researchers as a potential fluorescent detector for the efficient chiral recognition of α-phenylethylamine and tryptophan enantiomers [28]. Previous studies have shown that probes based on BINAM can highly selectively recognize lysine in aqueous solutions, but the results have indicated a significant fluorescent response for both configurations, thus being unable to distinguish the configuration of lysine [29]. This article reports the discovery and synthesis of an innovative fluorescent probe, which demonstrates exceptional selectivity for L-lysine under physiological conditions. The findings suggest that other amino acids do not interfere with its recognition capabilities. Moreover, the probe boasts advantages such as acid and alkali resistance, sensitivity, and prolonged recognition capacity. We have also investigated the influence of specific metal ions on the probe’s recognition effect and examined the substitution sites of the probe. The findings are detailed below.

## 2. Results and Discussion

### 2.1. Chiral Recognition of D/L-Lysine

We have designed and synthesized four novel fluorescent probes based on binaphthyl amine. These four probes have been found to be soluble in organic solvents and possess the ability to distinguish between D/L-lysine. Notably, this distinction is particularly pronounced in ethanol solutions. Subsequently, they were diluted into a PBS buffer solution (pH = 7.4) to serve as the liquid medium (as detailed in the Section 4).

**Figure 2:**
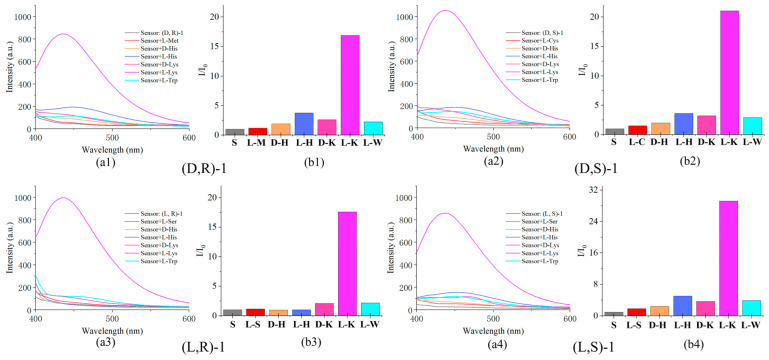
(**a**) Fluorescence spectra of several special amino acids (2 mM) with a probe (0.02 mM). (**b**) Plot of the fluorescence enhancement I/I_0_ at 437 nm. For ease of observation, we have only displayed representative data: S represents the blank group (sensor only) without the addition of any amino acids, with I/I_0_ = 1. The second item shows the highest I/I_0_ value among amino acids, excluding those presented. M stands for methionine, C for cysteine, and S for serine. The third and fourth items represent histidine (H), the fifth and sixth items represent lysine (K), and the seventh item represents tryptophan (W). The letters “D” and “L” before amino acid (or its abbreviation) represent chirality. In both Figure (**a**) and Figure (**b**) of each probe, the same substance is indicated by the same color.

To begin with, the fluorescence responses of these four probes toward 20 amino acids and their chiral forms were investigated. The comprehensive chart data can be found in Supplementary Materials Figures S1–S4. Upon excitation at a wavelength of 365 nm, it was observed that all four probes exhibited a significant enhancement in fluorescence specifically for L-lysine, as depicted in Figure 2a. Then, we used “I” to represent the fluorescence intensity of the probe in response to the component recognition, and “I_0_” denotes the initial fluorescence intensity of the probe. Therefore, the ratio I/I_0_ can be used as a direct measurement index for variations in fluorescence intensity, which reflects the result of the interaction between the probe and amino acids. A higher I/I_0_ value indicates a more significant enhancement of fluorescence. Figure 2b shows the I/I_0_ value at the maximum emission wavelength (437 nm) of the corresponding substance. The values of I/I_0_ for L-Lys were 16.89, 21.08, 17.60, and 29.23, respectively. In contrast, His and Trp exhibited values between 3.0 and 5.0, while the remaining amino acids ranged between 0.8 and 1.5. The chiral recognition efficacy, ef (ef = enantiomeric fluorescence enhancement ratio = [I_L_ − I_0_]/[I − I_0_] = ΔI_L_/ΔI_D_. I_0_, the fluorescence intensity of the probe. I_L_ or I_D_, fluorescence intensity with L or D substrate) was calculated as follows: ef [(D,R)-1] = 9.99, ef [(D,S)-1] = 9.12, ef [(L,R)-1] = 15.29 and ef [(L,S)-1] = 10.43, This demonstrates that all four probes are capable of efficiently and specifically recognizing L-Lys within chiral amino acids. The ef values of His and Trp are listed in Table S1 of the Supplementary Materials. The fluorescence emission spectrum of the (L,R)-1 blank group is shown in Figure S5.

Additionally, we conducted interference experiments to examine the effects of other amino acids on probe recognition. Upon examination, it was found that the presence of other amino acids had a minimal impact on the probe’s recognition efficacy. Consequently, this information has been relegated to Supplementary Materials Figures S6–S9.

### 2.2. Recognition in Different pH

**Figure 3:**
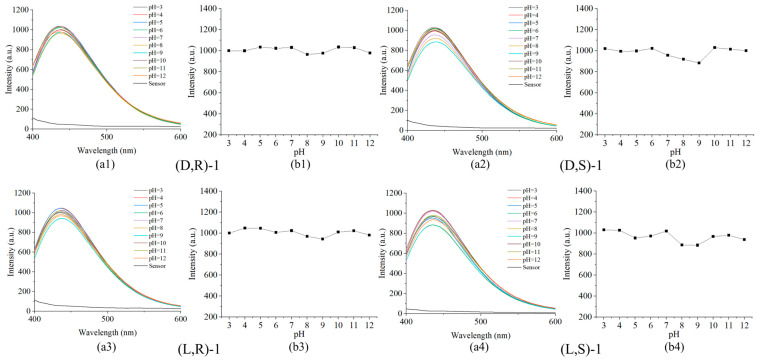
(**a**) Fluorescence spectra of probe (0.02 mM) with L-Lys (2 mM) with a pH range of 3 to 12, as well as the emission spectrum of the probe itself serving as a control. (**b**) Fluorescence Intensity Plot at 437 nm under Different pH Conditions.

We anticipate that these novel probes can be utilized in multiple fields, as the stability of the probe’s recognition of L-lysine across different pH levels is crucial for its widespread applicability. The results indicate that within the pH range of 3 to 12, the recognition efficiency of the probe for L-lysine is similar to its fluorescence intensity in PBS (pH = 7.4). Therefore, pH does not affect the probe’s ability to recognize L-lysine (Figure 3). The impact of pH on the luminescence spectrum of the probe is minimal, as demonstrated in Figure S10. Consequently, only the luminescence spectrum of the probe at a pH of 7 is presented in Figure 3. Similarly, the luminescence spectrum of D-Lys is displayed in Figure S11.

### 2.3. The Reaction Time of the Probes

**Figure 4:**
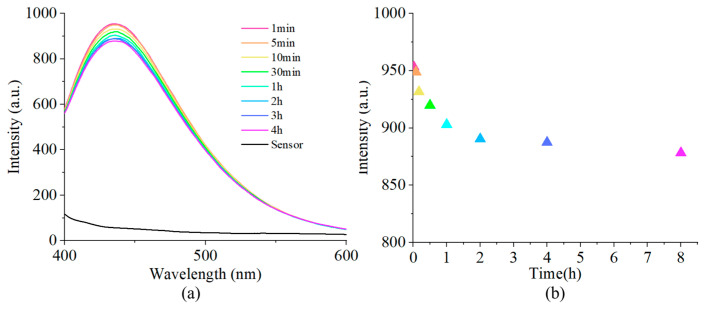
(**a**) Fluorescence spectra of probe (0.02 mM) with L-Lys (2 mM) at 1 min, 5 min, 10 min, 30 min, 1 h, 2 h, 4 h, and 8 h, as well as the emission spectrum of the probe itself serving as a control. (**b**) Plot of the fluorescence intensity at 437 nm.

Fluorescent probes typically require a rapid enhancement of fluorescence upon contact with the target substance, followed by a sustained emission over an extended period of time. Consequently, the reaction time between the probe and L-Lys has been investigated. Taking (L,R)-1 as an example, the study results show that the reaction between the probe and L-Lys is very rapid, allowing for recognition and detection within 1 min. The recognition effect of the probe at 8 h still retains 92.2% of its initial efficiency (Figure 4). Within one week, the fluorescence intensity was continuously monitored and no significant fluorescence quenching phenomenon was observed, as shown in Figure S12.

### 2.4. Limit of Detection

**Figure 5:**
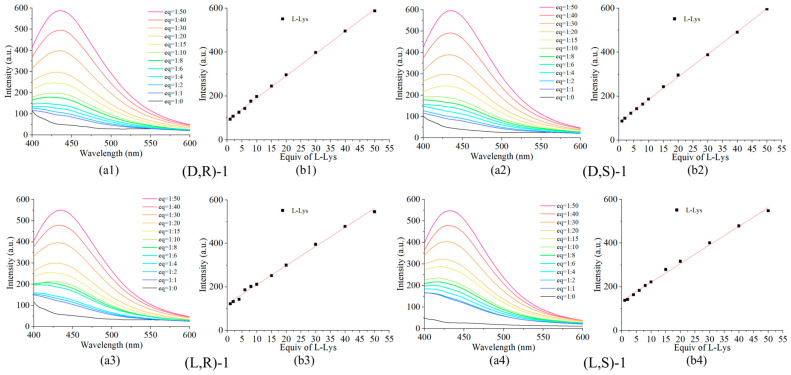
(**a**) Fluorescence spectra for the gradient recognition of 1 to 50 equivalents of L-Lys by the probe (0.02 mM) concentration, as well as the emission spectrum of the probe itself serving as a control. (**b**) Plot of the fluorescence intensity at 437 nm.

The study examined the fluorescence spectra of probes for L-lysine at different concentrations, as shown in Figure 5. The concentration of the probe was fixed at 0.02 mM, while the concentration of L-lysine ranged from 0.02 mM (eq = 1:1) to 1 mM (eq = 1:50). The fluorescence intensity significantly increased with the concentration of L-Lys, and the fluorescence intensity at λ = 437 nm was fitted using OriginPro 2021. The emission showed a linear positive correlation, with adjusted R-squared values for (D,R)-1, (D,S)-1, (L,R)-1, (L,S)-1 being 0.99883, 0.9994, 0.99455, 0.99536, respectively. Based on the limit of detection formula LOD = 3σ/K (where σ is the standard deviation of the probe samples, and K is the slope of the fitted line), the LOD for L-Lys using (D,R)-1, (D,S)-1, (L,R)-1, (L,S)-1 was calculated to be 2.12 × 10^−6^ M, 9.38 × 10^−6^ M, 5.98 × 10^−6^ M and 5.18 × 10^−6^ M. The data on the interaction between the probe and D-Lys can be found in Figure S13. We have also explored the LOD of (L,R)-1 under acidic and alkaline conditions (Figures S14–S16). The results indicate that at pH = 3, the LOD is 5.94 × 10^−6^ M, which is quite similar to the LOD at pH = 7.4 (5.98 × 10^−6^ M). However, when the pH increases to 11, the LOD rises to 8.07 × 10^−6^ M, slightly diminishing the identification performance. Nevertheless, these findings overall suggest that the probe exhibits sensitivity across a range of both acidic and alkaline environments.

### 2.5. The Effect of Metal Ions

**Figure 6:**
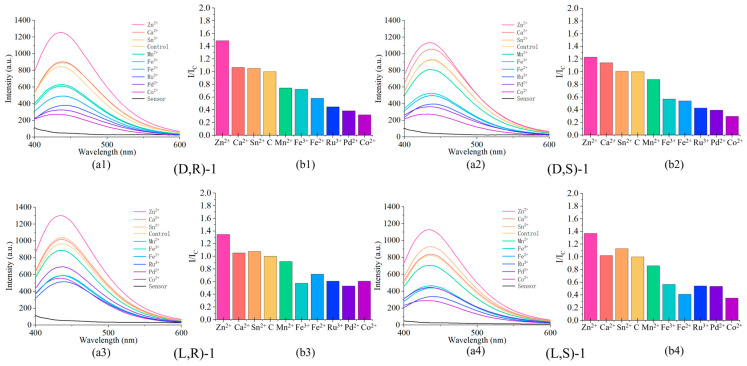
(**a**) Fluorescence spectrum upon addition of 2 equivalents of: Co^2+^, Fe^2+^, Fe^3+^, Mn^2+^, Pd^2+^, Ru^3+^, Zn^2+^, Sn^2+^, and Ca^2+^ to the probe, as well as the emission spectrum of the probe itself serving as a control. (**b**) Plot of the fluorescence intensity at 437 nm.

Metal ions can indeed form complexes that may impact the recognition effect of probes. In this study, the recognition effect of the probe on L-lysine was investigated in the presence of various metal ions: Co^2+^, Fe^2+^, Fe^3+^, Mn^2+^, Pd^2+^, Ru^3+^, Zn^2+^, Sn^2+^, and Ca^2+^. To ensure that anions did not interfere, all metal salts were chosen in their chloride forms. Among these metal ions, Zn^2+^ notably enhanced the probe’s recognition effect on L-Lys, showing an increase of at least 1.2 to 1.5 times compared to the control group. Sn^2+^ and Ca^2+^ also slightly enhanced the recognition effect (Figure 6). However, the presence of the remaining metal ions negatively impacted the probe’s ability to recognize L-Lys, suggesting interference or inhibition. Specifically, the presence of other metal ions, especially Co^2+^, Pd^2+^, Ru^3+^, Fe^2+^, and Fe^3+^, significantly reduced the probe’s recognition efficiency for L-Lys, with the recognition efficiency dropping to approximately half of its initial level. Fluorescent probes containing BINAM, BINAP, and BINOL structures often interact with Zn(II), which can be categorized into two main types: 1. Probes that form complexes with Zn(II) are used for the detection of other substances [27,30,31,32,33]; 2. The probe we designed, which contains the BINAM structure. The probe directly recognizes Zn(II), which leads to a change in fluorescence [34,35,36,37]. The probe we designed contains the BINAM structure, and thus, it may also exhibit a specific recognition effect towards Zn(II), potentially resulting in an enhancement of fluorescence. The data on the interaction between the probe and D-Lys can be found in Figure S17.

### 2.6. Confirm the Reaction Site by ^19^F NMR

**Figure 7:**
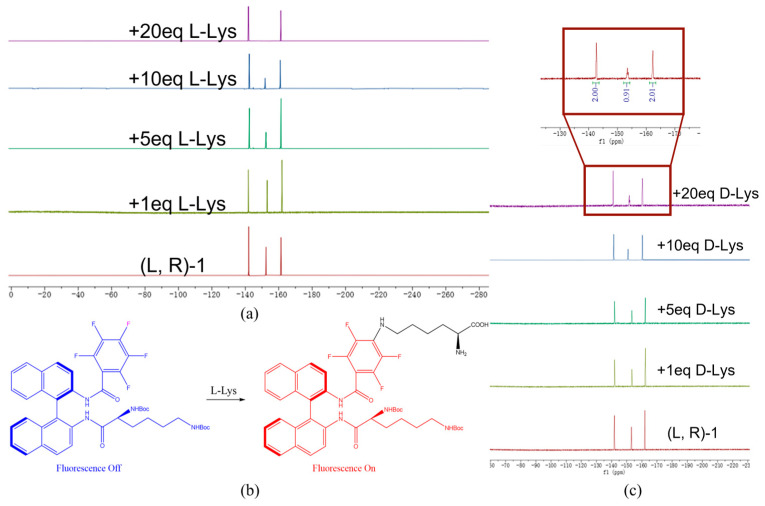
(**a**) ^19^F NMR spectra of (L,R)-1 treated with 0–20 equivalents of L-Lys. (**b**) (L,R)-1 showed significant fluorescence enhancement for L-Lys through nucleophilic substitution. (**c**) ^19^F NMR spectra of (L,R)-1 treated with 0–20 equivalents of D-Lys.

The reaction between (L,R)-1 and L-Lys was investigated utilizing ^19^F NMR spectroscopy. Figure 7a presents the ^19^F NMR spectra of (L,R)-1 treated with 0–20 equivalents of L-Lys (DMSO:D_2_O = 3:1). Three different fluorines on the phenyl group of (L,R)-1 exhibited peaks at δ-142.2, -152.6, and -161.4, with a 2:1:2 intensity ratio. The signal at δ-152.6 corresponds to the para-fluorine. With the addition of L-Lys, the signal at δ-152.6 weakened, and upon increasing Lys to 20 equivalents, the signal at δ-152.6 completely disappeared. This indicates that the para-fluorine atom of (L,R)-1 is nucleophilically substituted by L-Lys, showing high regioselectivity. Figure 7b presents a possible binding mechanism between (L,R)-1 and L-lysine. Figure 7c shows the ^19^F NMR titration experiment of (L,R)-1 reacting with D-lysine as a comparison, where the peak area ratio of the three groups is 2.00:0.91:2.01 after adding 20 times D-lysine. Consequently, the probe exhibits poor reactivity with D-lysine. The ε-amino group of L-lysine interacts with the para-positioned F atom of the probe. Steric hindrance and hydrogen bonding may be the reasons why D-lysine does not react well with the probe molecule.

**Figure 8:**
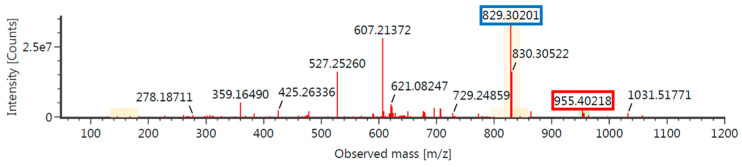
MS spectra of (L,R)-1 mixed with L-Lys. The value 829.30201 represents the probe combined with Na^+^, while 955.40218 indicates the new molecule formed after the reaction between the probe and L-Lys, also combined with Na^+^.

The mass spectrometry of (L,R)-1 mixed with L-lysine also confirmed the appearance of new molecules produced by recognition, as shown in Figure 8. The exact molecular weight of the probe calculated by ChemDraw is 806.31028. The mass spectrometry data show that its *m*/*z* value after binding with Na^+^ is 829.30201 and subtracting Na^+^ (22.98977) gives the measured *m*/*z* value as 806.31224. Similarly, the molecular *m*/*z* calculated value of the probe after reaction with L-lysine is 932.40958, the *m*/*z* value after binding with Na^+^ is 955.40218, and the new measured molecular *m*/*z* value is 932.41241.

### 2.7. Quantum Yield

**Figure 9:**
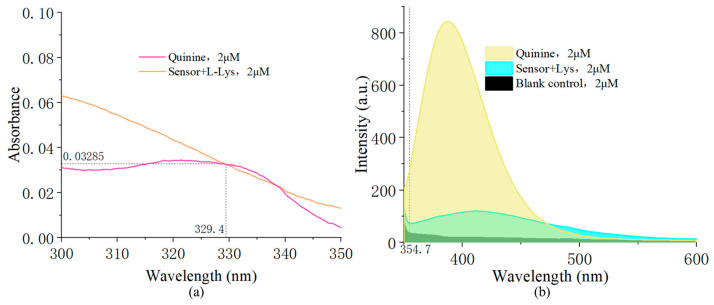
(**a**) Absorption spectrum of (L,R)-1 and quinine sulfate between 300 nm and 350 nm. (**b**) Emission spectrum of (L,R)-1 (2 µM) + L-Lys, Quinine Sulfate (2 µM), and Blank Solvent from 350 to 600 nm.

To investigate the fluorescence efficiency of a probe, it is necessary to conduct experiments to measure the fluorescence quantum yield. The UV absorption spectra of the (L,R)-1 blank group as well as after adding L-lysine are presented in Figure S18. The absorbance of sulfate quinine and (L,R)-1 at a wavelength of 329 nm within a concentration range of 2–10 μM exhibits a linear relationship with their concentrations, as depicted in Figures S19 and S20. This means that within this concentration range, the absorbance can be approximately considered to be proportional to the concentration. The 2 μm concentration was selected as the concentration for determining fluorescence quantum yield. The experimental steps are based on the literature reported by Levitus [38]. Figure 9a shows that there are two intersections in the absorption spectrum of (L,R)-1 and quinine sulfate between 300 nm and 350 nm. A wavelength of 329 nm was chosen for excitation, at which point the absorbance was 0.03285, which is less than 0.04. Calculate the quantum yield using the following formula, where *X* represents the test sample, *S* the reference material, *Q* the fluorescence quantum yield, *E* the integrated fluorescence intensity, *A* the solution absorbance, and *n* the solution refractive index.

$$Q_{X}=Q_{S}\cdot \frac{A_{S}}{A_{X}}\cdot \frac{E_{X}}{E_{S}}\cdot \frac{n_{X}^{2}}{n_{S}^{2}}$$

Supplementary Material Figure S21 shows the fluorescence enhancement of quinine sulfate starting at 350.2 nm, and Figure 9b presents the fluorescence enhancement of the probe beginning at 354.7 nm. The literature provides a quantum yield of 0.527 for quinine sulfate [39]; thus, during the detection of L-Lysine, the calculated fluorescence quantum yield of (L,R)-1 is 0.117, which may have a certain degree of error. Figure S22 illustrates the quantum yield of the probe itself, which is merely 0.023. Consequently, it can be inferred that the probe barely emits light on its own.

## 3. Materials and Methods

### 3.1. Reagents and Apparatus

^1^H, ^13^C, and ^19^F NMR spectra were measured using a Bruker AM600 NMR spectrometer (BRUKER, Bellingham, MA, USA). The chemical shifts of the NMR spectra are given in ppm relative to the internal reference TMS (1H, 0.00 ppm) for protons. HRMS spectral data were recorded on a Bruker Micro-TOF-QII mass spectrometer (BRUKER, Bellingham, MA, USA). Fluorescence spectra were obtained at 298 K using a (F97 Pro) spectrofluorophotometer (Shanghai Lengguang Technology Co., Ltd., Shanghai, China) with an excitation wavelength of 365 nm. Melting point determination is performed using the SGW S-4A micro melting point apparatus, produced by Shanghai INESA Physico-Optical Instrument Co., Ltd. (Shanghai, China). All chemicals involved in the experiments were purchased from Adamas Chemistry Co., Ltd. (Shanghai, China). The chemicals were obtained from commercial suppliers and used without further purification. All solvents used in the optical spectroscopy studies were of HPLC or spectroscopic grade.

### 3.2. Synthesis of L-Lys-Boc_2_

**Scheme 1:**
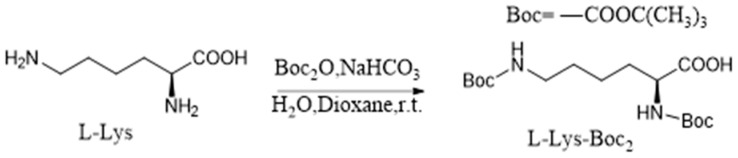
Synthesis of L-Lys-Boc_2_.

L-lysine (731 mg, 5 mmol) was added to 100 mL of H_2_O containing NaHCO_3_ (840 mg, 10 mmol), and, under stirring, Boc_2_O (1500 mg, 6.87 mmol) dissolved in 50 mL of dioxane was slowly added, followed by stirring at room temperature for 12 h. Boc_2_O (1500 mg, 6.87 mmol) dissolved in 50 mL of dioxane was then added slowly again under stirring, and the reaction progress was monitored by thin-layer chromatography (TLC). After the reaction proceeded for an additional 12 h, it was extracted three times with 50 mL of DCM and washed three times with 20 mL of H_2_O. The organic solvent was removed under reduced pressure, and the crude product was purified by silica gel column chromatography, eluting with DCM/MeOH (30:1) to obtain a yellow gel-like L-Lys-Boc_2_ with a yield of 84% (1455 mg, 4.2 mmol), as shown in Scheme 1. Similarly, a colorless gel-like D-Lys-Boc_2_ was obtained using the same method, with a yield of 80%.

### 3.3. Synthesis of (L,R)-1

**Scheme 2:**
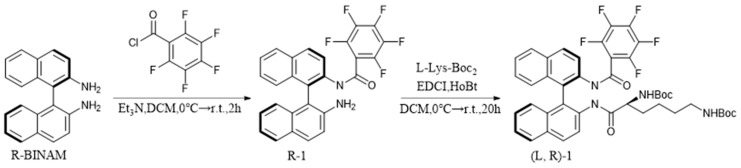
Synthesis of (L,R)-1.

To a solution of R-BINAM (284 mg, 1 mmol) in DCM (30 mL), triethylamine (200 μL, 1.5 mmol) was added. After cooling the mixture to 0 °C, 2,3,4,5,6-pentafluorobenzoyl chloride (230 mg, 1 mmol) was slowly added dropwise, and the mixture was stirred at room temperature for 8 h. The progress of the reaction was monitored by thin-layer chromatography (TLC). After the reaction was complete, the mixture was washed three times with 20 mL of H_2_O. The organic solvent was removed under reduced pressure, and the crude product was purified by silica gel column chromatography, eluting with DCM/PE (1:1) to obtain a pale-yellow powder R-1, with a yield of 65% (310 mg, 0.65 mmol). The same method yielded a pale-yellow powder S-1, with a yield of 59%. Next, R-1 (239 mg, 0.5 mmol) was added to a DCM solution (30 mL) and stirred at 0 °C. After cooling the system to 0 °C, HOBt (81 mg, 0.6 mmol) and EDCI (93 mg, 0.6 mmol) were added, and the mixture was stirred for 2 min before adding L-Lys-Boc_2_ (346 mg, 1 mmol). The system was then transferred to room temperature and stirred for 20 h, with the progress of the reaction monitored by TLC. After the reaction was complete, the mixture was washed three times with 20 mL of H_2_O. On the back of removing the organic solvent under reduced pressure, the crude product was purified by silica gel column chromatography, eluting with DCM/MeOH (50:1), and then the organic solvent was removed under reduced pressure; a white powder defined as (L,R)-1 was obtained, with a yield of 34% (137 mg, 0.17 mmol), as shown in Scheme 2. L/D is the configuration of Lys-Boc_2_ and R/S is the configuration of BINAM. The same method yielded (L,S)-1 with a yield of 32%, (D,R)-1 with a yield of 38%, and (D,S)-1 with a yield of 28%.

(L,R)-1: **^1^H NMR (400 MHz, DMSO-*d*_6_)** δ = 0.65–1.04 (m, 6H), 1.38 (s, 18H), 2.57–2.77 (m, 2H), 2.82–2.94 (m, 1H), 6.60–6.80 (m, 2H), 6.92–7.04 (m, 2H), 7.23–7.34 (m, 2H), 7.42–7.55 (m, 2H), 7.69–7.89 (m, 2H), 7.97–8.11 (m, 3H), 8.13–8.21 (m, 1H), 9.06 (s, 1H), 9.93 (s, 1H). **^13^C NMR (100 MHz, CHLOROFORM-*d*_6_)** δ = 21.8, 28.3, 28.4, 28.5, 29.2, 29.7, 29.8, 30.3, 31.5, 31.7, 32.0, 39.5, 53.5, 54.4, 79.3, 80.2, 123.3, 123.4, 125.3, 125.3, 125.5, 126.1, 126.3, 126.3, 127.4, 127.5, 128.3, 128.5, 129.6, 130.3, 132.1, 132.2, 132.4, 132.7, 133.7, 133.9, 155.7, 156.2, 172.2. **^19^F NMR (376 MHz, DMSO-*d*_6_)** δ = −161.55–−160.88 (m), −152.43 (t, *J* = 22.1 Hz), −142.48 to −141.62 (m). **HRMS(ESI+)** *m*/*z* calcd for C_43_H_43_F_5_N_4_O_6_ [M + Na]^+^: 806.31028, found 806.31117. **m.p**: 74 °C.

(L,S)-1: **^1^H NMR (400 MHz, DMSO-*d*_6_)** δ = 0.75–1.00 (m, 6H), 1.38 (s, 18H), 2.57–2.75 (m, 2H), 2.86–2.89 (m, 1H), 6.53–6.78 (m, 2H), 6.91–7.02 (m, 2H), 7.22–7.29 (m, 2H), 7.41–7.54 (m, 2H), 7.81–7.88 (m, 1H), 7.92–8.10 (m, 4H), 8.10–8.20 (m, 1H), 8.70 (s, 1H), 10.20 (s, 1H). **^13^C NMR (100 MHz, CHLOROFORM-*d*_6_)** δ = 22.2, 22.5, 28.2, 28.4, 28.5, 29.5, 29.8, 30.9, 32.4, 40.1, 52.4, 53.3, 53.5, 55.6, 79.2, 80.7, 123.6, 125.2, 125.6, 125.9, 126.6, 127.4, 127.6, 128.4, 130.0, 130.3, 132.2, 132.5, 133.5, 156.2, 173.4. **^19^F NMR (376 MHz, DMSO-*d*_6_)** δ = −162.19–−160.43 (m), −152.66 (t, *J* = 22.2 Hz), −141.93 (dd, *J* = 24.9, 8.1 Hz). **HRMS(ESI+)** *m*/*z* calcd for C_43_H_43_F_5_N_4_O_6_ [M + Na]^+^: 806.31028, found 806.31109. **m.p**: 74 °C.

(D,R)-1: **^1^H NMR (400 MHz, DMSO-*d*_6_)** δ = 0.69–1.00 (m, 6H), 1.38 (s, 18H), 2.57–2.75 (m, 2H), 2.80–2.96 (m, 1H), 6.57–6.81 (m, 2H), 6.92–7.04 (m, 2H), 7.23–7.33 (m, 2H), 7.40–7.55 (m, 2H), 7.82–7.87 (m, 1H), 7.94–8.12 (m, 4H), 8.14–8.21 (m, 1H), 9.06 (s, 1H), 9.93 (s, 1H). **^13^C NMR (100 MHz, CHLOROFORM-*d*_6_)** δ = 22.2, 22.4, 28.1, 28.1, 28.4, 28.5, 29.5, 31.3, 32.6, 39.7, 40.2, 52.4, 53.5, 53.8, 55.2, 77.4, 79.2, 79.7, 80.1, 81.9, 122.2, 123.8, 125.2, 125.7, 125.8, 126.6, 127.3, 127.6, 128.4, 128.4, 130.0, 130.2, 131.6, 132.3, 132.6, 133.5, 134.3, 145.4, 155.6, 156.2, 156.9, 171.5. **^19^F NMR (376 MHz, DMSO-*d*_6_)** δ = −162.19 to −160.09 (m), −152.34 (dt, *J* = 61.7, 22.3 Hz), −142.15 (dd, *J* = 65.2, 16.8 Hz). **HRMS(ESI+)** *m*/*z* calcd for C_43_H_43_F_5_N_4_O_6_ [M + Na]^+^: 806.31028, found 806.31146. **m.p**: 89 °C.

(D,S)-1: **^1^H NMR (400 MHz, DMSO-*d*_6_)** δ = 0.69–1.09 (m, 6H), 1.38 (s, 18H), 2.60–2.73 (m, 2H), 2.82–2.93 (m, 1H), 6.55–6.82 (m, 2H), 6.91–7.03 (m, 2H), 7.23–7.33 (m, 2H), 7.40–7.55 (m, 2H), 7.81–7.88 (m, 1H), 7.95–8.12 (m, 4H), 8.12–8.21 (m, 1H), 8.70 (s, 1H), 10.21 (s, 1H). **^13^C NMR (100 MHz, CHLOROFORM-*d*_6_)** δ = 22.2, 22.4, 28.1, 28.1, 28.4, 28.5, 29.5, 29.8, 31.3, 32.6, 39.7, 52.4, 53.5, 53.8, 55.2, 79.2, 80.1, 81.9, 122.2, 123.0, 123.8, 125.2, 125.7, 125.8, 126.6, 127.3, 127.5, 128.3, 128.4, 130.0, 130.1, 131.6, 132.3, 132.6, 133.5, 134.3, 138.8, 142.8, 145.4, 155.6, 156.2, 156.9, 171.5. **^19^F NMR (376 MHz, DMSO-*d*_6_)** δ = −161.37 (td, *J* = 24.8, 24.2, 8.1 Hz), −152.41 (t, *J* = 22.1 Hz), −142.16–−141.70 (m). **HRMS(ESI+)** *m*/*z* calcd for C_43_H_43_F_5_N_4_O_6_ [M + Na]^+^: 806.31028, found 806.31037. **m.p**: 89 °C.

The reproducibility experiment is shown in Figure S23, with data taken from 10 groups. This demonstrates the replicability of the experiment. The four fluorescent probes were characterized by ^1^H NMR, ^13^C NMR, ^19^F NMR, and MS, with the spectral data provided in the Supplementary Materials Figures S24–S28.

### 3.4. Preparation of Samples for Fluorescence Analysis

Solutions of four sensors were separately prepared at a concentration of 2.0 mM in ethanol, and amino acids were prepared at a concentration of 200 mM in Deionized water as stock solutions, to be used immediately upon preparation. In the study of selectivity towards amino acids, 30 μL of the sensor/EtOH stock solution was mixed with 30 μL of the amino acid/H_2_O stock solution, left to stand for 10 min, and then diluted with PBS buffer (pH = 7.4) to 3000 μL to form the detection solution. In the study of amino acids’ selectivity, 30 μL of the L-Lys stock solution was first mixed with 30 μL of the stock solution of another amino acid, left to stand for 10 min, followed by the addition of 30 μL of the sensor/EtOH stock solution, and then diluted with PBS buffer to 3000 μL to form the detection solution. For studying the recognition in different pHs, 30 μL of the sensor/EtOH stock solution was mixed with 30 μL of the Lys/H_2_O stock solution, left to stand for 10 min, and then diluted with PBS buffer at the corresponding pH to 3000 μL to form the detection solution, adjusting the pH with dilute solutions of NaOH and HCl. In the study of the detection limit of the probes, Lys was re-prepared at a concentration of 20 mM in Deionized water as the stock solution. Then, 30 μL of the sensor/EtOH stock solution was mixed with 3× μL of the Lys/H_2_O stock solution, left to stand for 10 min, and then diluted with buffer to 3000 μL to form the detection solution, where x corresponds to the concentration ratio of Lys. In the study of the effect of metal ions on probe recognition, 30 μL of the sensor/EtOH stock solution was first mixed with 30 μL of a 4.0 mM solution of metal chloride and left to stand for 10 min, followed by the addition of 30 μL of the L-Lys stock solution; then, it was diluted with PBS buffer at pH 7.4 to 3000 μL to form the detection solution.

In the experiments described, the volume concentration of EtOH in the detection solution is 1%, and the concentration of the sensor is 0.02 mM. Except for the experiments determining the detection limit, the concentration of amino acids is maintained at 2 mM. All fluorescence data, except for those related to stability studies, were obtained within 3 h. Unless specifically stated, the data were obtained at room temperature with PBS buffer (pH = 7.4, EtOH/PBS = 1/99, *v*/*v*) solution as the solvent. Reaction time: 30 min. PMT: 650 V. Error bars from three independent experiments. λexc = 365 nm. Slit: 20/10 nm.

## 4. Conclusions

In summary, we discovered a set of BINAM-based fluorescent probes capable of chiral recognition of L-lysine among natural amino acids. The recognition effect is not influenced by other amino acids, and the probe is stable under certain acidic and alkaline conditions, allowing for rapid identification and long-term stability. The probe is sensitive and can identify L-Lys at lower concentrations. The recognition effect of the probe is enhanced in the presence of zinc ions. We also verified through ^19^F NMR that the para-fluorine atom of the probe undergoes nucleophilic substitution with L-Lys. The quantum yield has also been determined. This work provides a novel, efficient, and convenient method for the chiral recognition of L-Lys.

## Data Availability

Data is contained within the article or Supplementary Materials.

## Supplementary Materials

The following supporting information can be downloaded at: https://www.mdpi.com/article/10.3390/ijms25147504/s1.
