# Supplementary material for: Spectroscopic Study of a Novel Binaphthyl Amine Fluorescent Probe for Chiral Recognition of D/L-Lysine
Source: Int J Mol Sci. 2024 Jul 9;25(14):7504. doi: 10.3390/ijms25147504 (PMC11277325; doi:10.3390/ijms25147504)
Supplement: Supplementary file 1 [file ijms-25-07504-s001.zip › ijms-3058457-supplementary.pdf]

Supplementary Materials of

# Spectroscopic Study of a Novel Binaphthyl Amine Fluorescent Probe for Chiral Recognition of D/L-Lysine

**Figures S1-S4：** (a) Fluorescence spectra of probe (0.02 mM) to various amino acids (2 mM). (b) Plot of the fluorescence enhancement $I/I_0$ at 437 nm. The sequence of amino acids tested includes: D/L-Ala(A), D/L-Arg(R), D/L-Asn(N), D/L-Asp(P), D/L-Cys(C), D/L-Gln(Q), D/L-Glu(E), Gly(G), D/L-His(H), D/L-Iso(I), D/L-Leu(L), D/L-Lys(K), D/L-Met(M), D/L-Phe(F), D/L-Pro(P), D/L-Ser(S), D/L-Thr(T), D/L-Trp(W), D/L-Tyr(Y) ,and D/L-Val(V).

The values of $I/I_0$ for L-Lys were 16.89, 21.08, 17.60 and 29.23, respectively. In contrast, His and Trp exhibited values between 3.0-5.0, while the remaining amino acids ranged between 0.8 and 1.5.

In Figure (a), only the data with significant fluorescence enhancement has been marked, while in Figure (b), the fluorescence intensities of all amino acids at 437 nm (the maximum emission wavelength) are presented.

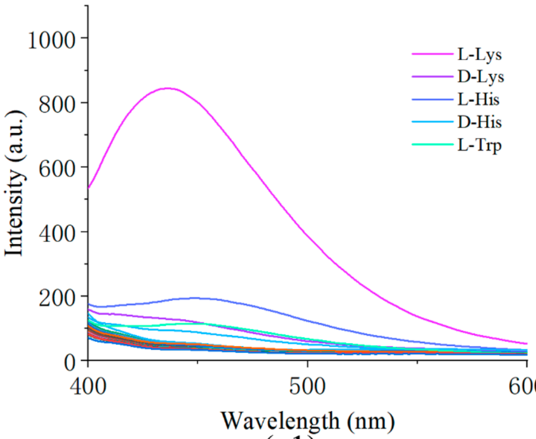


(a1)

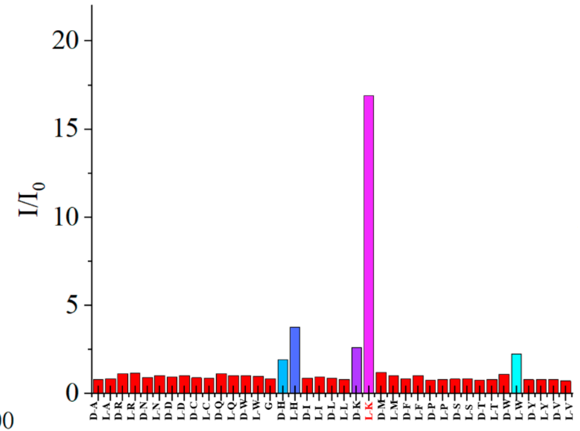


(D,R)-1

(b1)

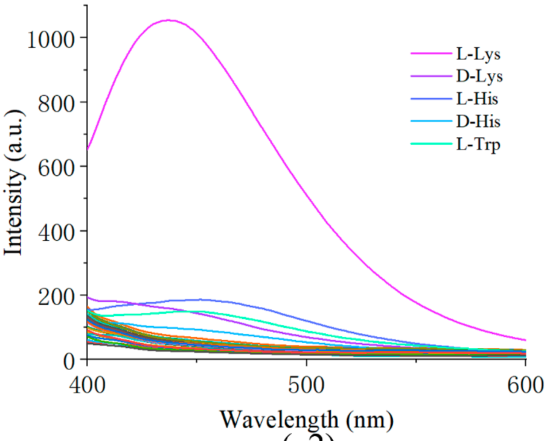


(a2)

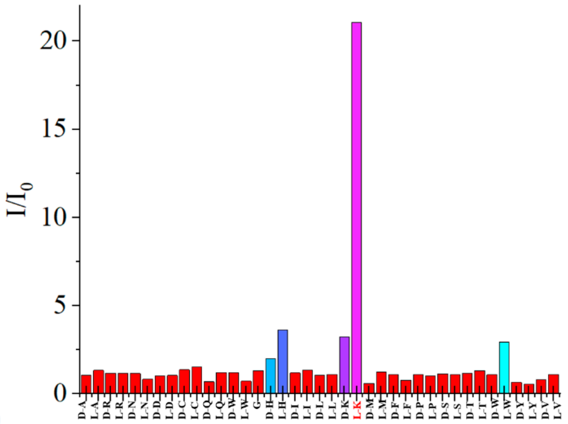


(D,S)-1

(b2)

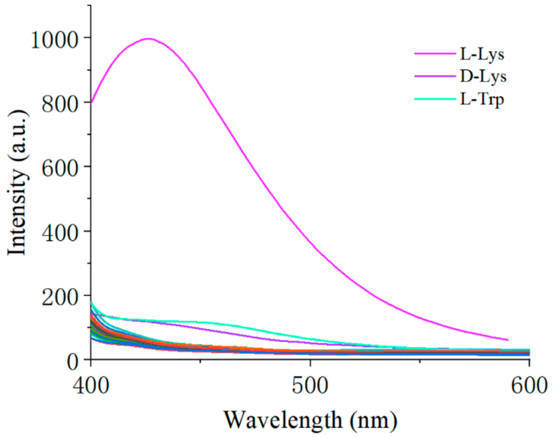


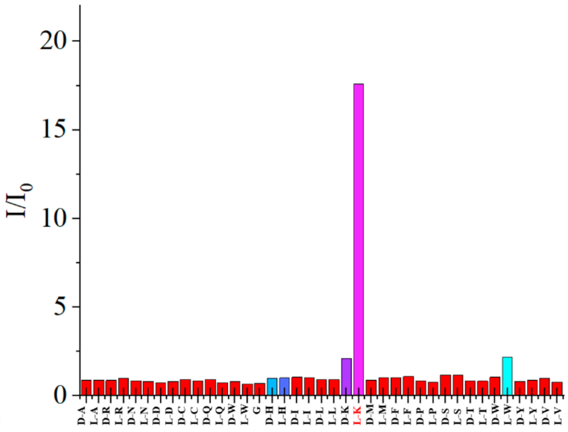


(a3) (L,R)-1 (b3)

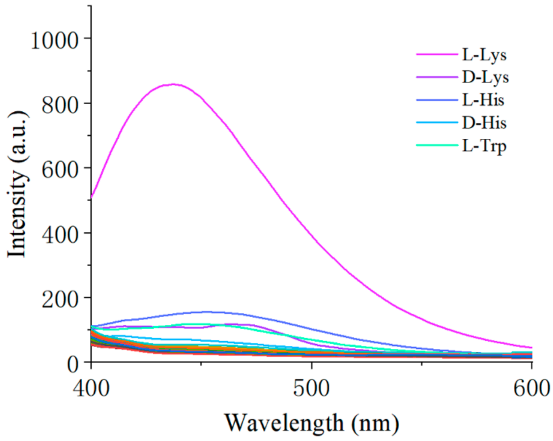


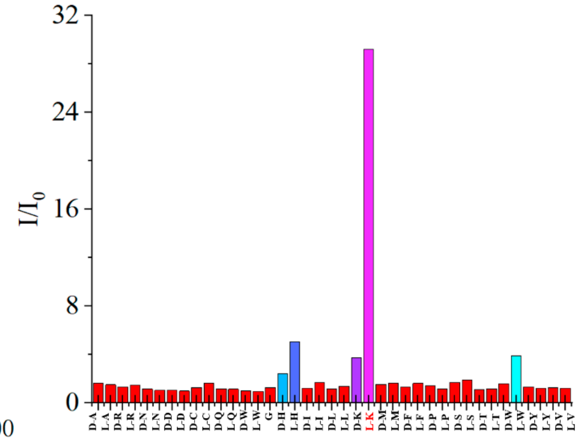


(a4) (L,S)-1 (b4)

**Figure S5:** Fluorescence spectra of (L, R)-1 blank group at a concentration of 2μM, as well as the groups containing 100-fold equivalent amounts of D-Lys and L-Lys.

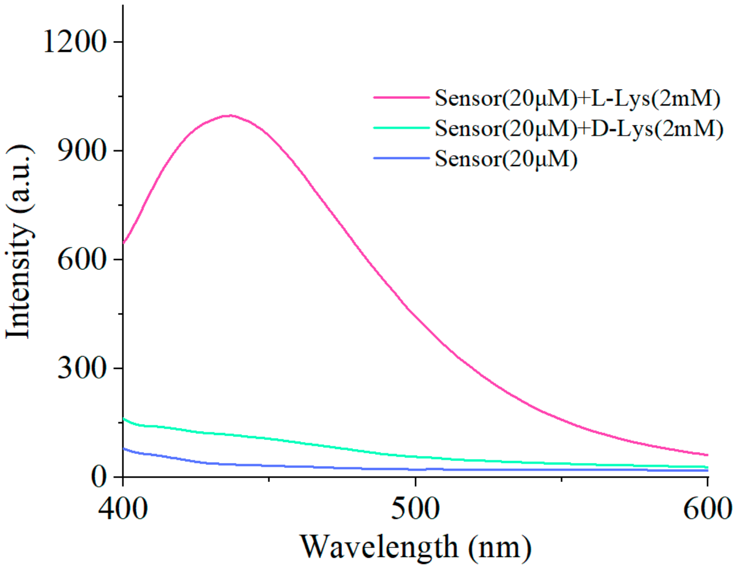

**Table S1**: The method of calculating the enhancement factor (ef) values through the ratio of $I/I_0$ and the ef values for three amino acids are presented. The fluorescence intensity of amino acids that do not react with the probe is close to the fluorescence intensity of the probe itself, hence calculating the ef value is meaningless. Notably, the four probes do not recognize D-Trp, making the calculation of the ef value for Trp highly erroneous. Additionally, (L, R)-1 does not recognize D/L-His, thus the highlighted five values are meaningless.

$$ef = \frac{\Delta I_L}{\Delta I_D} = \frac{I_L - I_0}{I_D - I_0} = \frac{\frac{I_L - I_0}{I_0}}{\frac{I_D - I_0}{I_0}} = \frac{\frac{I_L}{I_0} - 1}{\frac{I_D}{I_0} - 1}$$

| | (D, R)-1 | (D, S)-1 | (L, R)-1 | (L, S)-1 |
|---|---|---|---|---|
| Lys | 9.97 | 9.12 | 15.29 | 10.43 |
| His | 3.03 | 2.66 | 0.15 | 2.82 |
| Trp | 14.45 | 24.18 | 24.77 | 4.97 |

**Figures S6–S9**: Interference by other amino acids in probe recognition

In living organisms, multiple amino acids are simultaneously present. It is crucial to study the recognition of chiral lysine in the presence of various amino acids for newly synthesized probes. Under physiological conditions (PBS buffer, pH=7.4), the fluorescence absorption intensity at 437nm was measured for the other 19 amino acids and their chiral forms, each at the same concentration as L-lysine. Strong intensity peaks were observed for all of them (**Figures S6-S9. a**). To compare this interference, we set "$I_C$" as the fluorescence intensity when the probe recognizes L-lysine and "I" as the fluorescence intensity when the probe recognizes L-lysine in the presence of other amino acids. Our research results indicate that all values of $I/I_C$ range between 0.9 and 1.1. Therefore, in the presence of other amino acids, the probe can effectively recognize L-lysine (**Figures S6-S9. b**).

(a) Fluorescence spectra of probe (0.02mM) with L-Lys (2mM) in the presence of other amino acids (2mM). (b) Plot of the fluorescence enhancement I/Ic at 437 nm. The sequence of amino acids tested includes: Control Group(C), D/L-Ala(A), D/L-Arg(R), D/L-Asn(N), D/L-Asp(P), D/L-Cys(C), D/L-Gln(Q), D/L-Glu(E), Gly(G), D/L-His(H), D/L-Iso(I), D/L-Leu(L), D-Lys(K), D/L-Met(M), D/L-Phe(F), D/L-Pro(P), D/L-Ser(S), D/L-Thr(T), D/L-Trp(W), D/L-Tyr(Y) ,and D/L-Val(V).

Since no amino acids have been found that significantly affect the probe's recognition of L-Lysine, Figure (a) only labels the data when the probe recognizes L-Lysine alone without the addition of any interfering amino acids (Control). In contrast, Figure (b) presents the fluorescence intensity at 437 nm (the maximum emission wavelength) after the addition of other interfering amino acids.

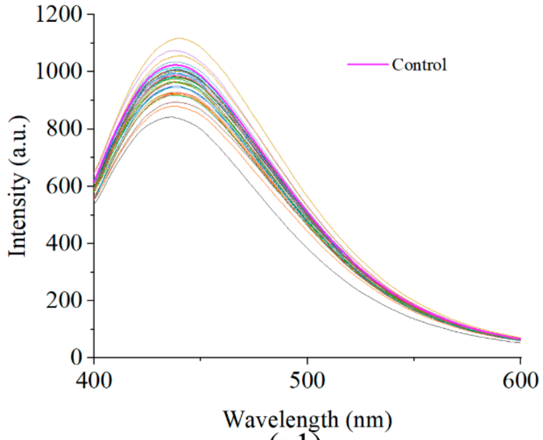


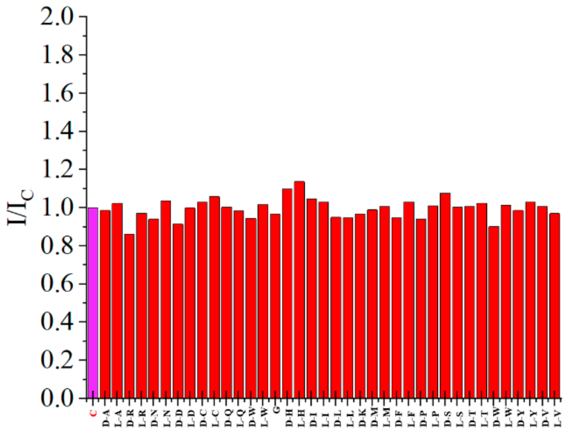


(a1) (D,R)-1 (b1)

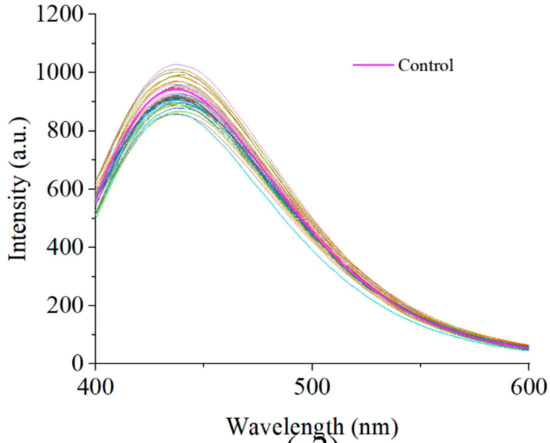


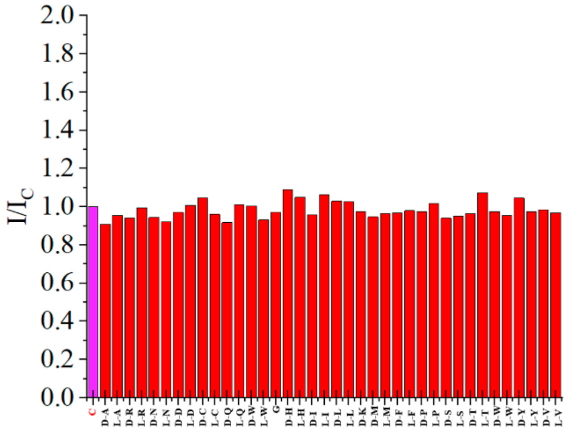


(a2) (D,S)-1 (b2)

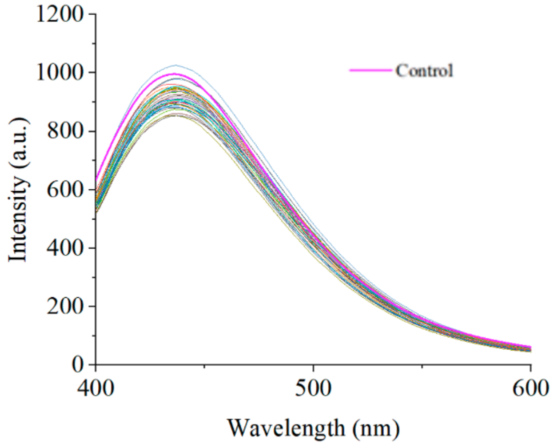


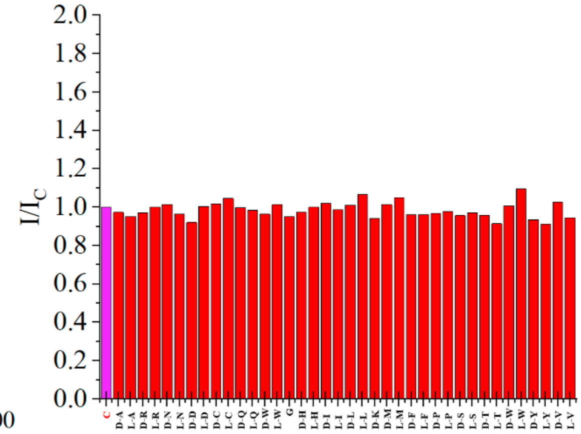


(a3) (L,R)-1 (b3)

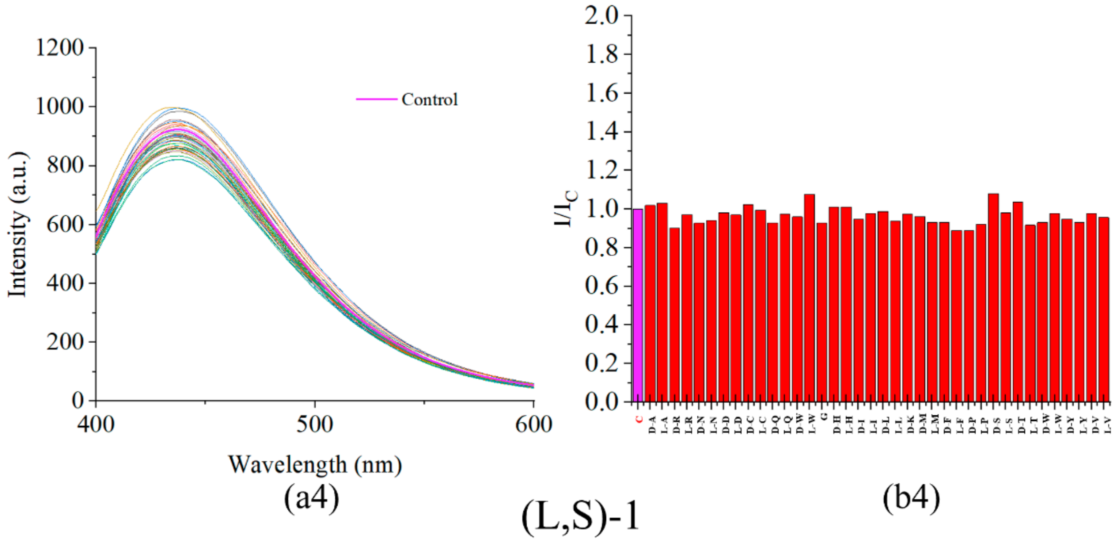


**Figure S10:** The luminescent spectra of the probe were detected under room temperature in PBS buffer(1% V/V EtOH) solution with pH values of 3, 5, 7, 9, and 11.

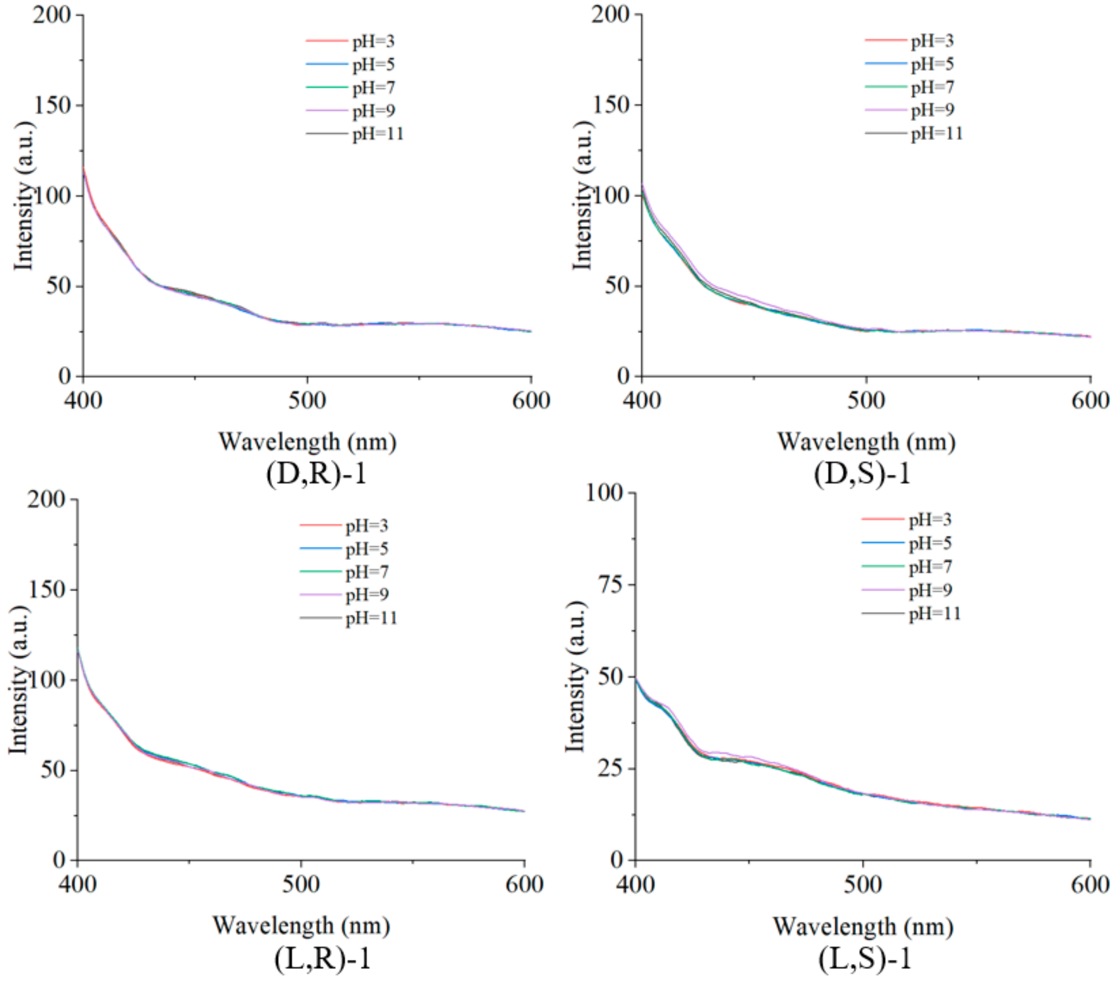

**Figure S11**: At room temperature, in PBS buffer (1% V/V EtOH) with a pH of 3-12, the fluorescent response of (D/L, R/S)-1 (0.02 mM) upon recognizing 100 equivalents of D-Lys is observed. The probe still does not recognize D-Lys after changing the pH.

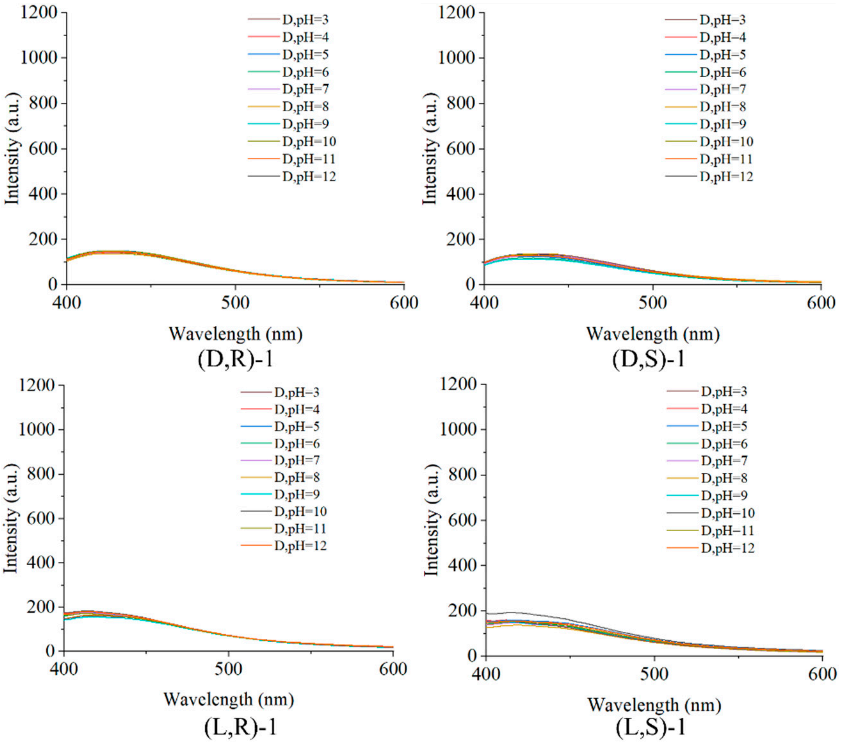


**Figure S12**: Fluorescence intensity variation within a week.

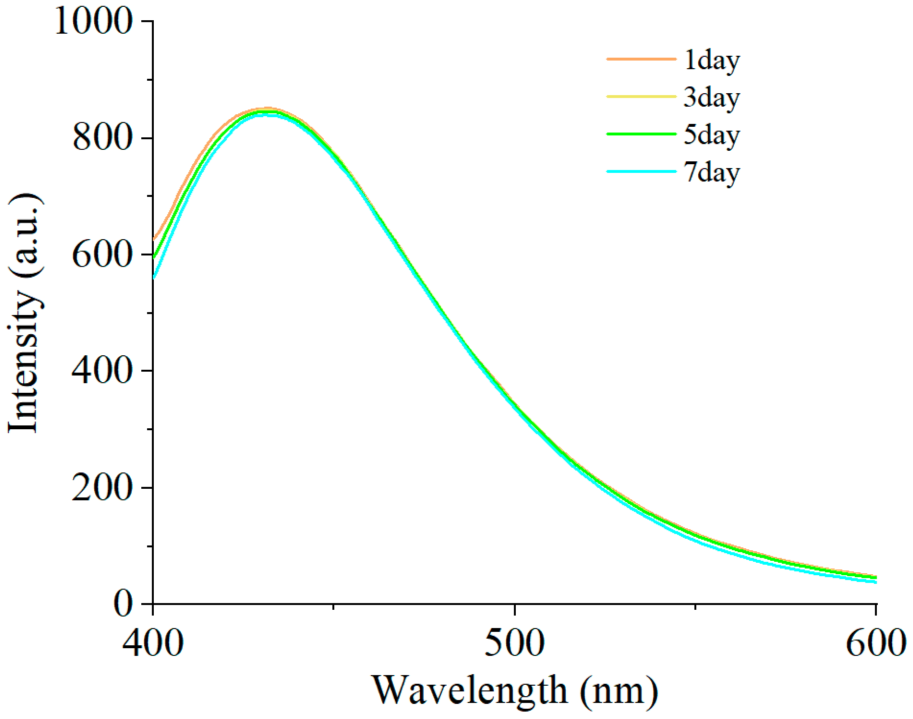

**Figure S13:** In PBS buffer (pH=7.4, 1% V/V EtOH), the fluorescence response of (D/L, R/S)-1 (0.02 mM) to the concentration gradient recognition of 1 to 50 equivalents of D-Lys is observed. After changing the concentration, the probe still does not recognize D-Lys.

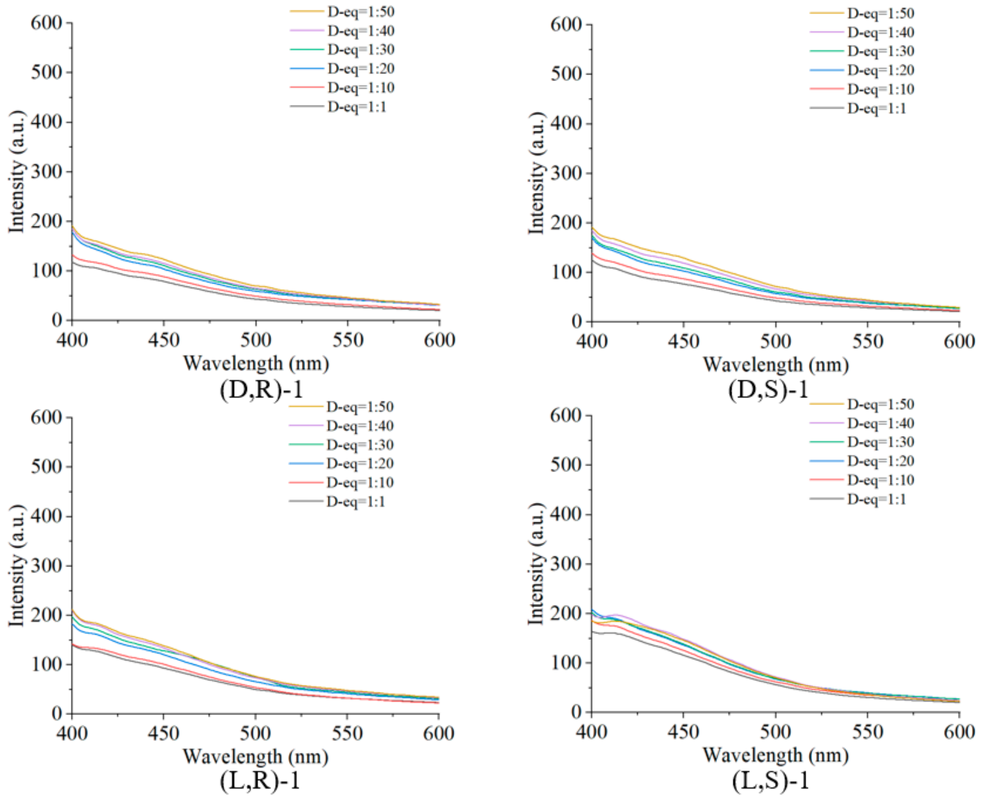


**Figure S14:** (a) Fluorescence spectra for the gradient recognition of 1 to 50 equivalents of L-Lys by the probe (0.02 mM) concentration at pH=3.(b) Plot of the fluorescence intensity at 436 nm.

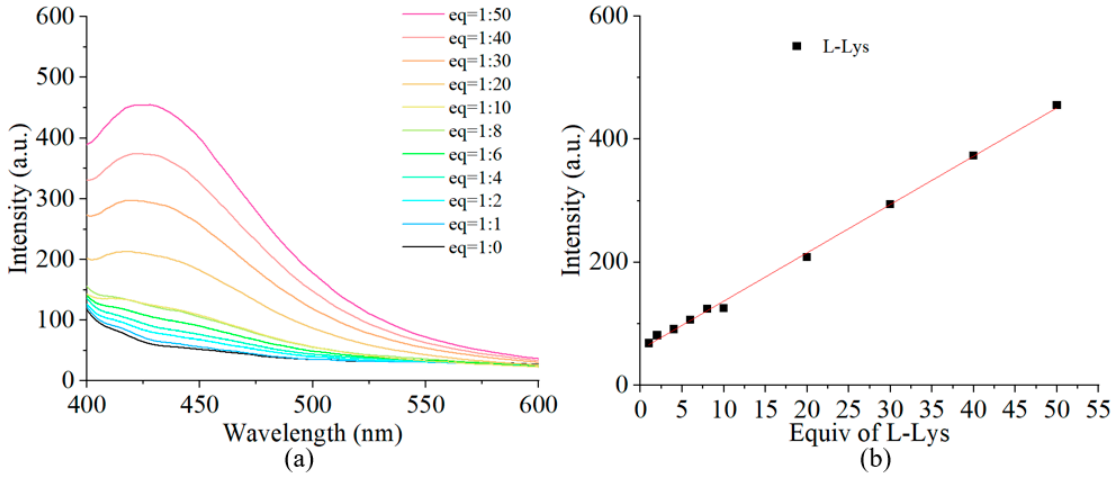

**Figure S15**：(a) Fluorescence spectra for the gradient recognition of 1 to 50 equivalents of L-Lys by the probe (0.02 mM) concentration at pH=11. (b) Plot of the fluorescence intensity at 436 nm.

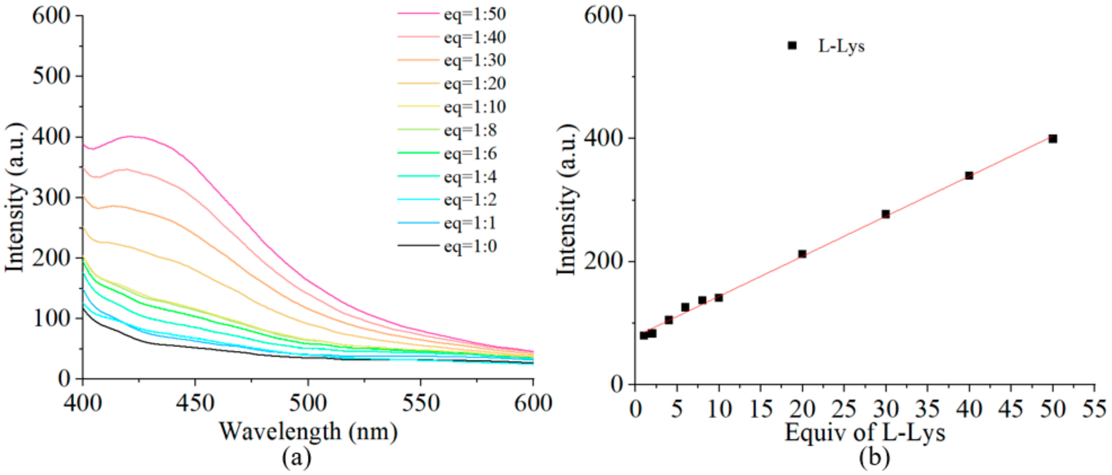


**Figure S16**：Comparison of fitting lines at pH=3 and pH=11.

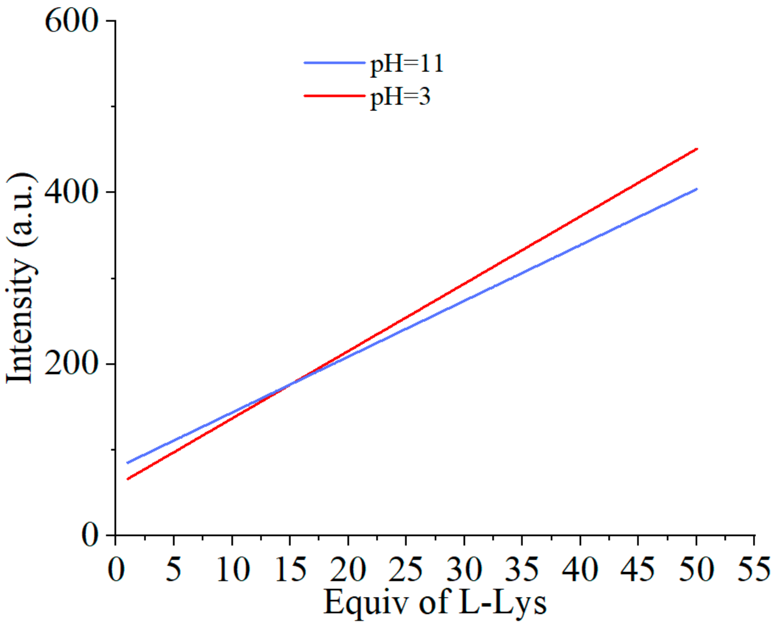

**Figure S17:** In PBS buffer (pH 7.4, 1% V/V EtOH), upon the addition of 2 equivalents of metal ions $Co^{2+}$, $Fe^{2+}$, $Fe^{3+}$, $Mn^{2+}$, $Pd^{2+}$, $Ru^{3+}$, $Zn^{2+}$, $Sn^{2+}$, $Ca^{2+}$ to (D/L, R/S)-1 (0.02 mM), the fluorescence response when recognizing 100 equivalents of D-Lys is observed. Even after the addition of metal ions, the probe still does not recognize D-Lys.

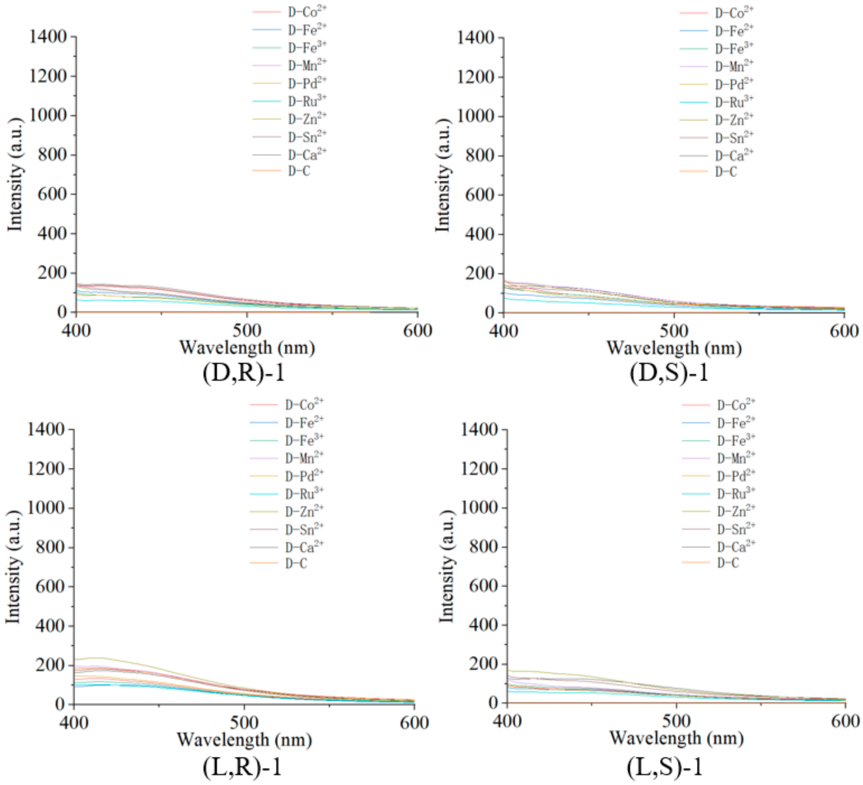


**Figure S18:** UV absorption spectra under working conditions of (L, R)-1 as well as absorption spectra at the same concentration without adding L-lysine.

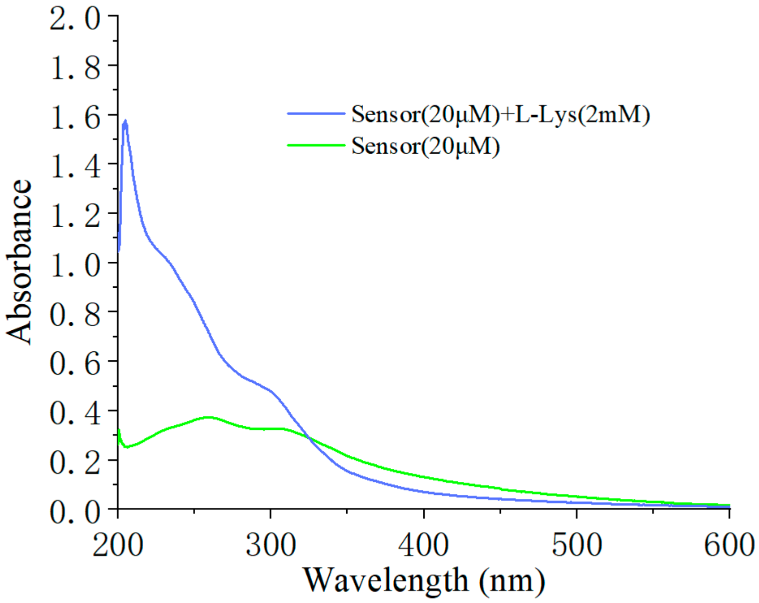

**Figure S19:** (a) UV absorption spectra of sulfate quinine at concentrations of 2μM, 2μM, 6μM, 8μM, and 10μM. Solvent: 1N $H_2SO_4$.（b）Absorbance intensity plot at 329 nm. The adjusted R-squared of the linear fitting equation is 0.990.

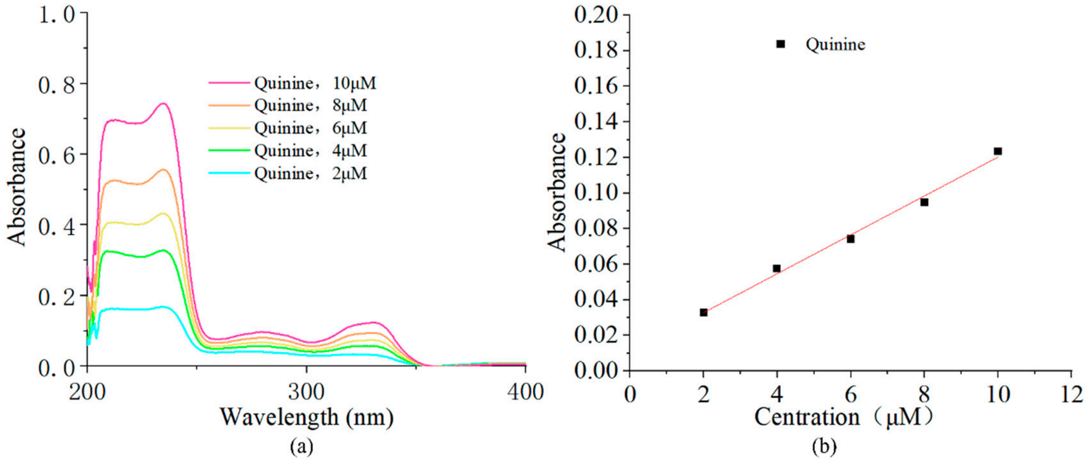


**Figure S20:** (a) UV absorption spectra of (L, R)-1 at concentrations of 2μM, 2μM, 6μM, 8μM, and 10μM. The concentration of L-lysine in each sample is 100 times that of the probe. Solvent: 1% EtOH (v/v) in PBS, pH=7.4.（b）Absorbance intensity plot at 329 nm. The adjusted R-squared of the linear fitting equation is 0.981.

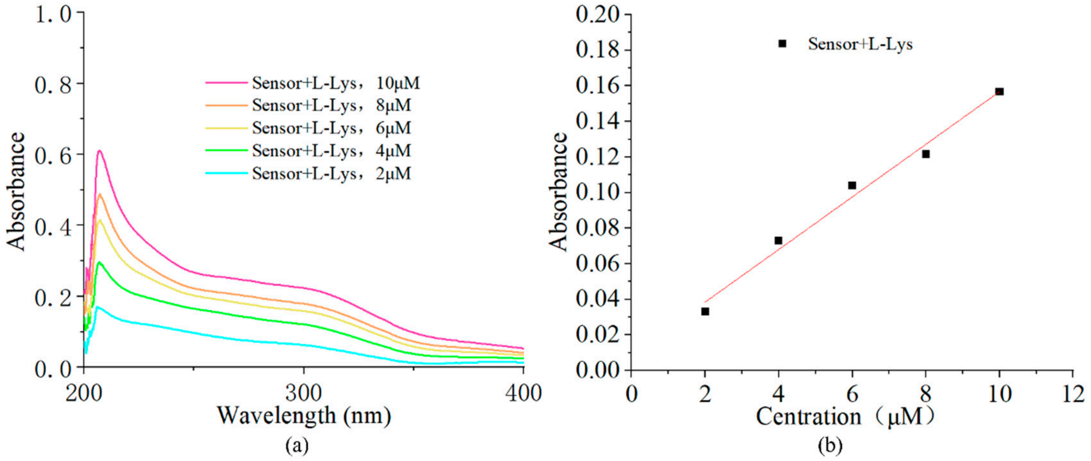

**Figure S21：** The fluorescence spectrum of quinine sulfate excited at 329 nm.

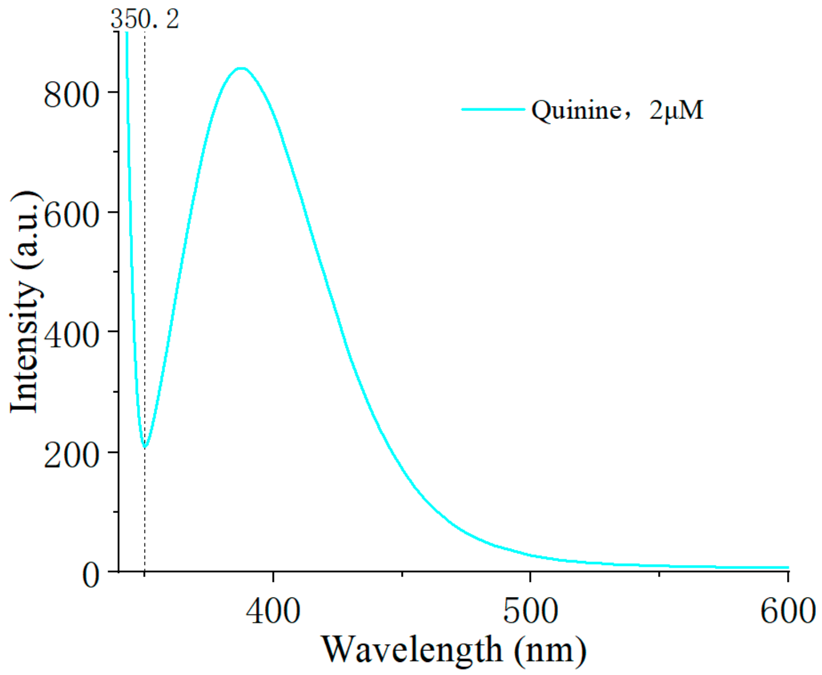


**Figure S22：** Emission Spectrum of (L,R)-1(2μM), Quinine Sulfate(2μM), and Blank Solvent from 350-600 nm.

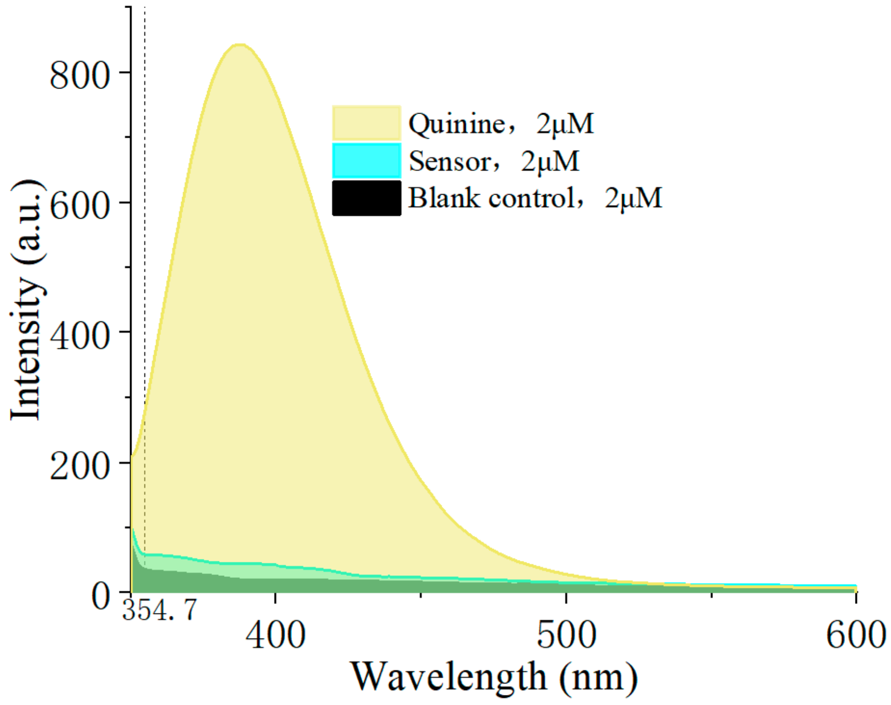

**Figure S23:** Repetitive Experiments: Fluorescence intensity measurements were conducted on eight sets of solutions with identical concentrations, and the results demonstrated excellent reproducibility of the experiments. (a) Fluorescence spectra of (L, R)-1 at eight groups with a concentration of 2 μM each, tested against 20 mM D-Lys and L-Lys.(b) Graph of fluorescence emission intensity at 437 nm.

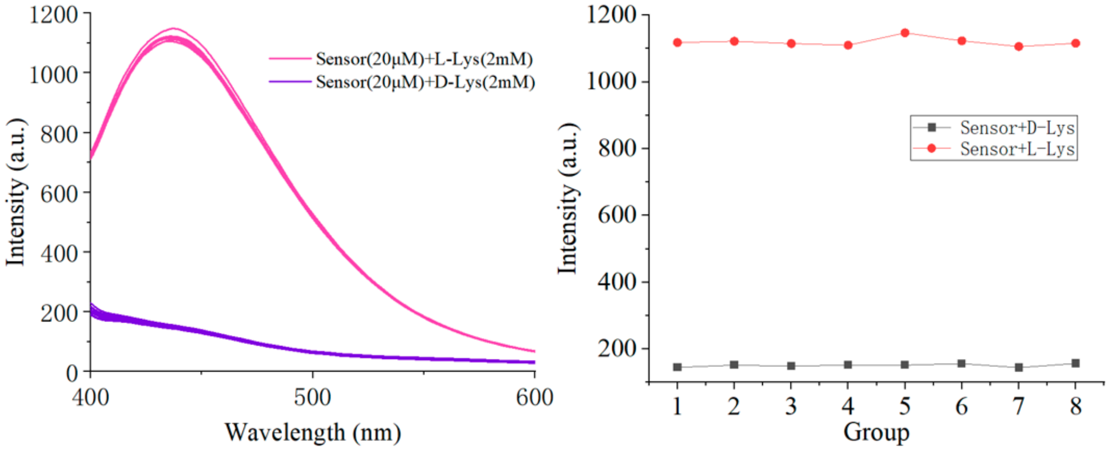


**Figure S24:** $^{1}$H NMR spectra of R/S-1.（400M DMSO）

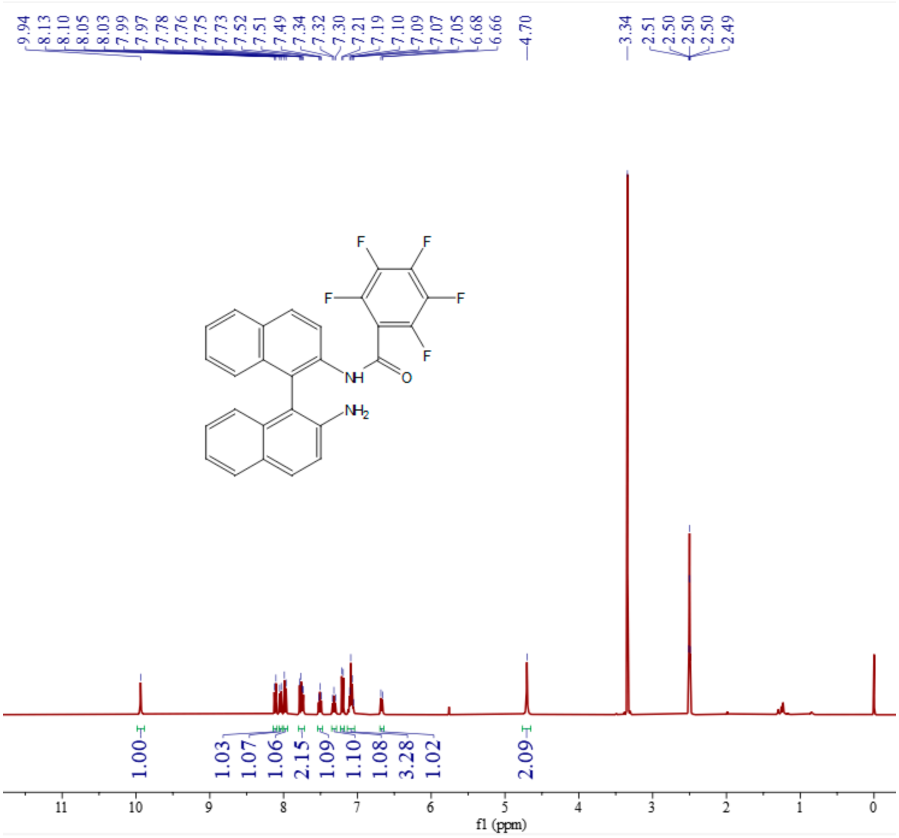

**Figure S25:** $^{1}$H NMR spectra of (D/L，R/S)-1.（400M DMSO）

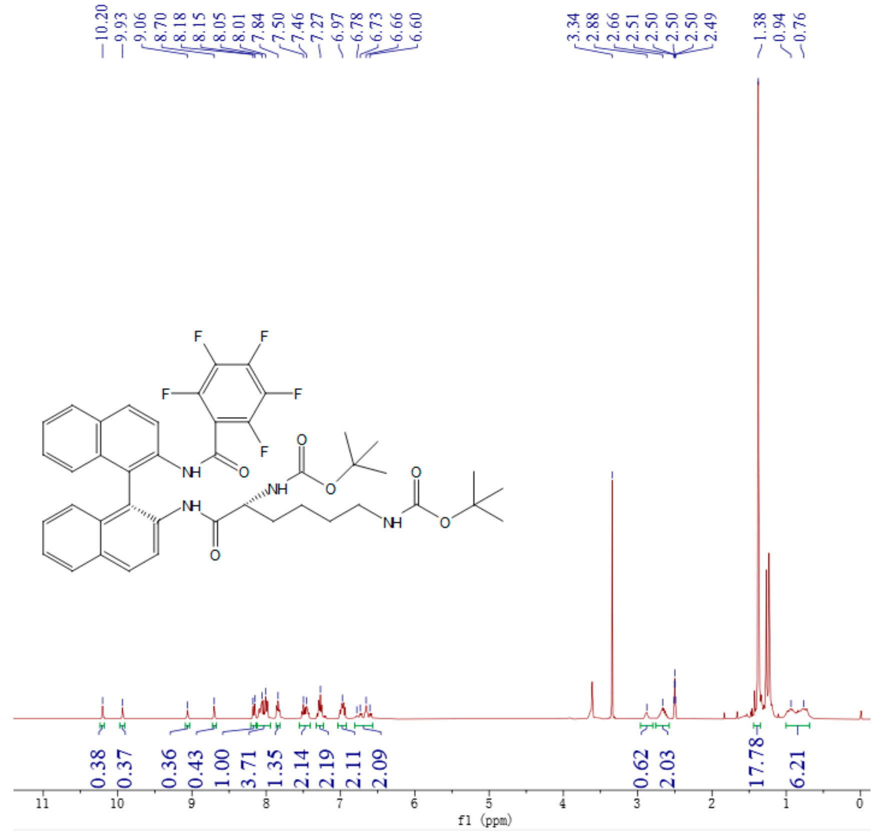

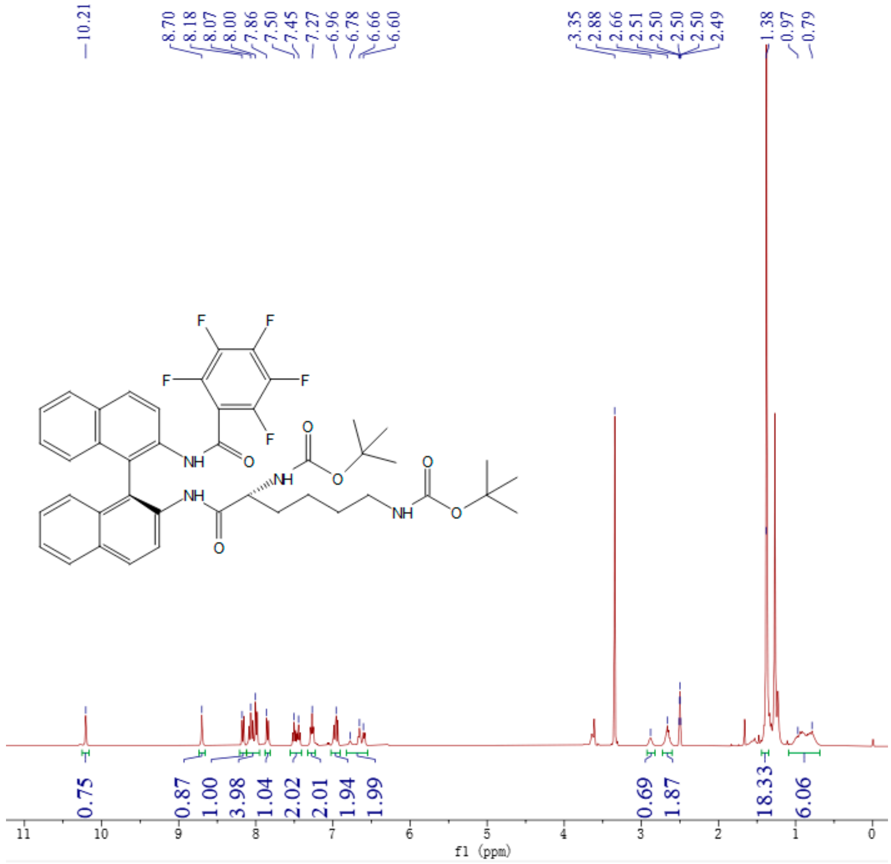

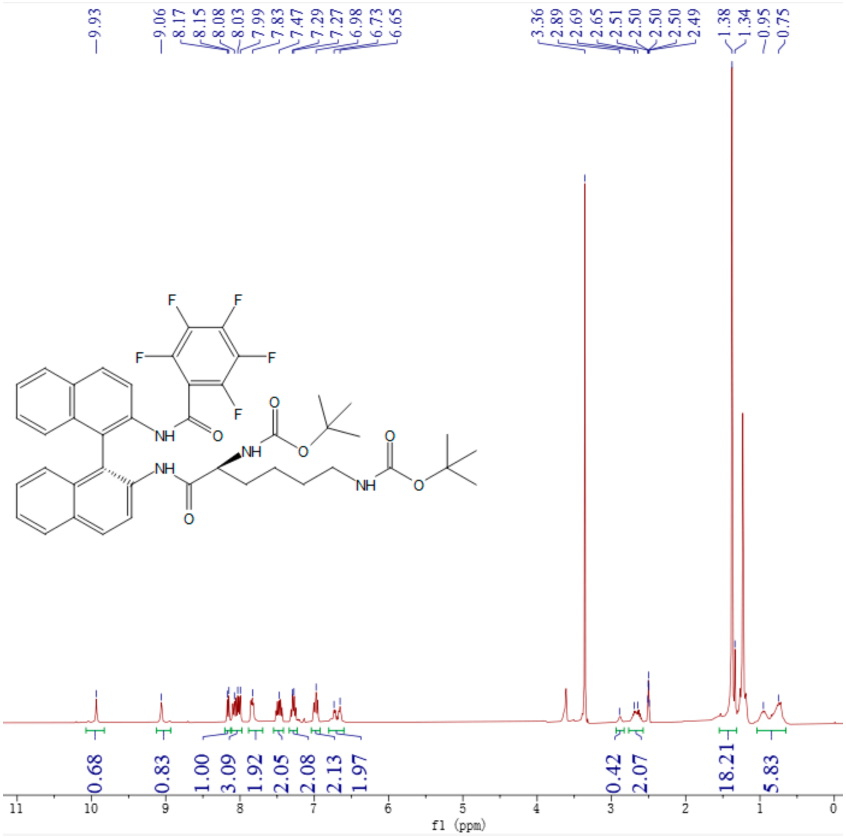
f1 (ppm)

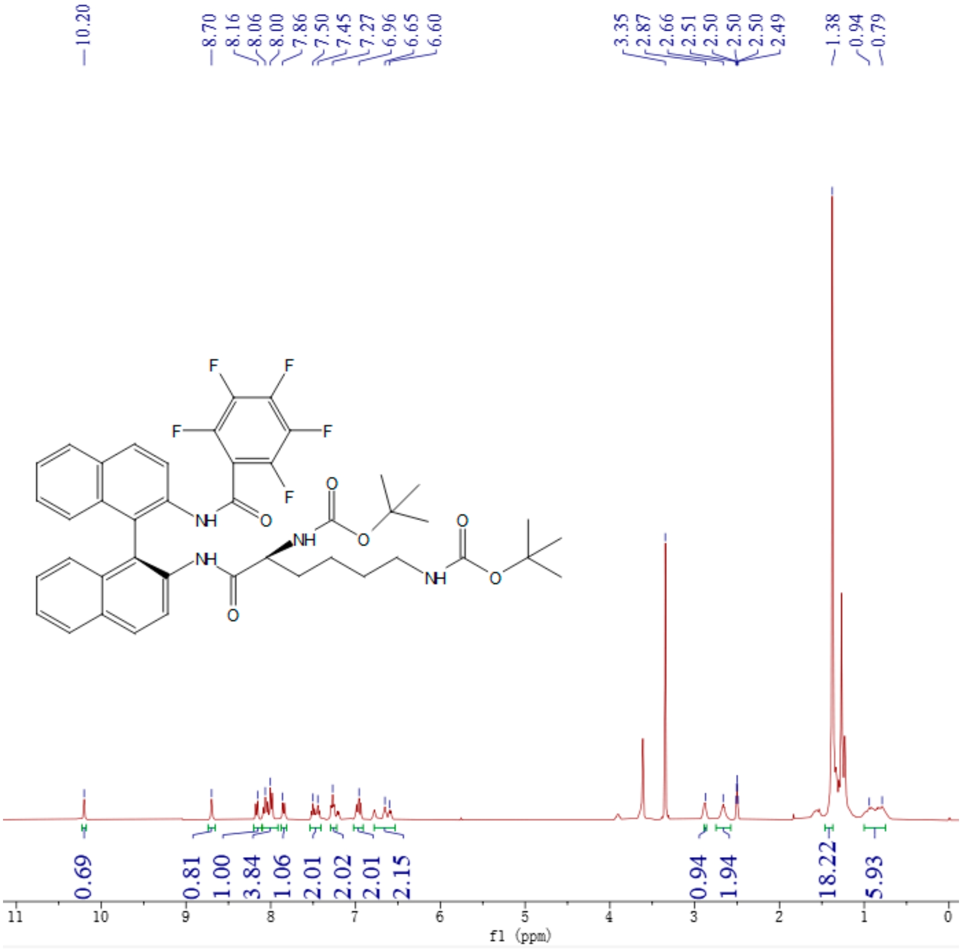
f1 (ppm)

**Figure S26:** $^{13}$C NMR spectra of (D/L，R/S)-1.（400M DMSO）

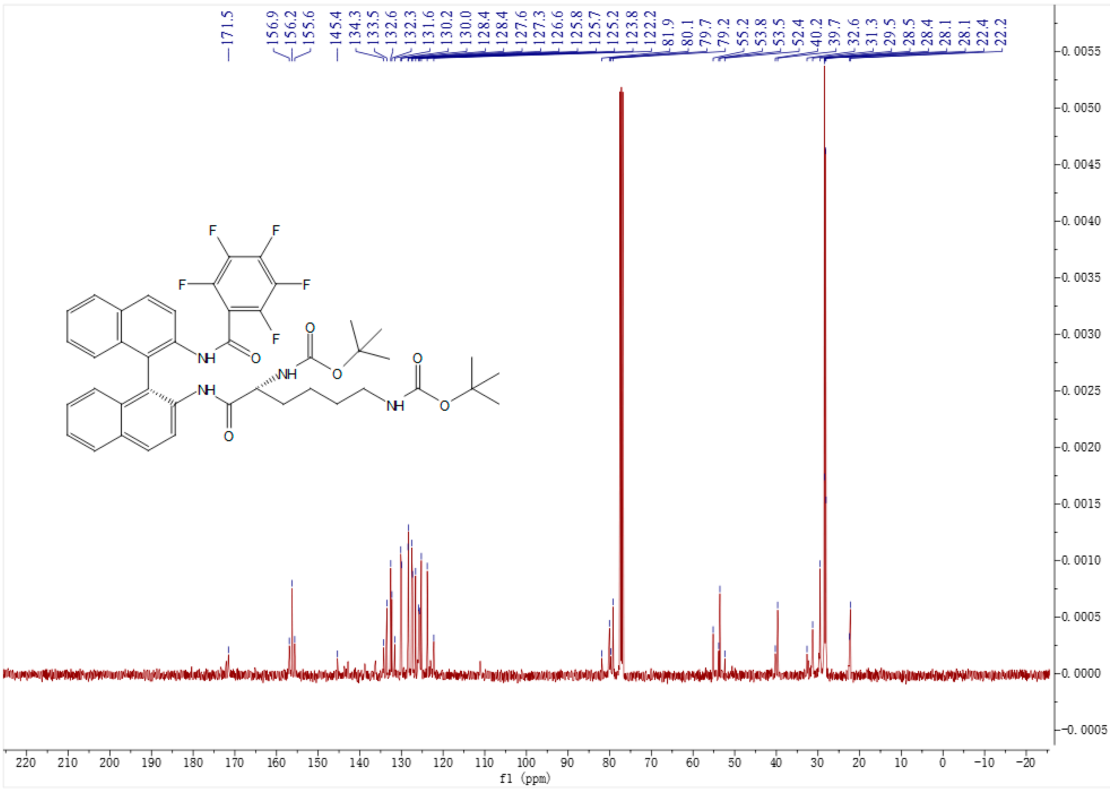

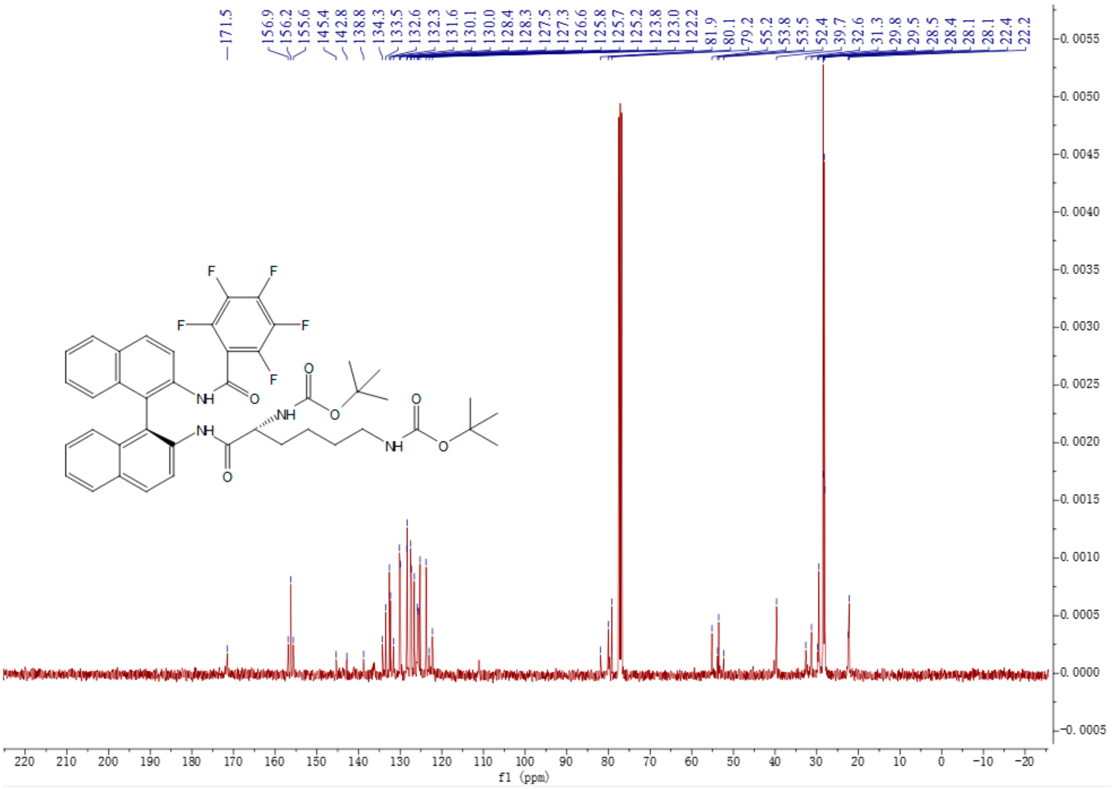

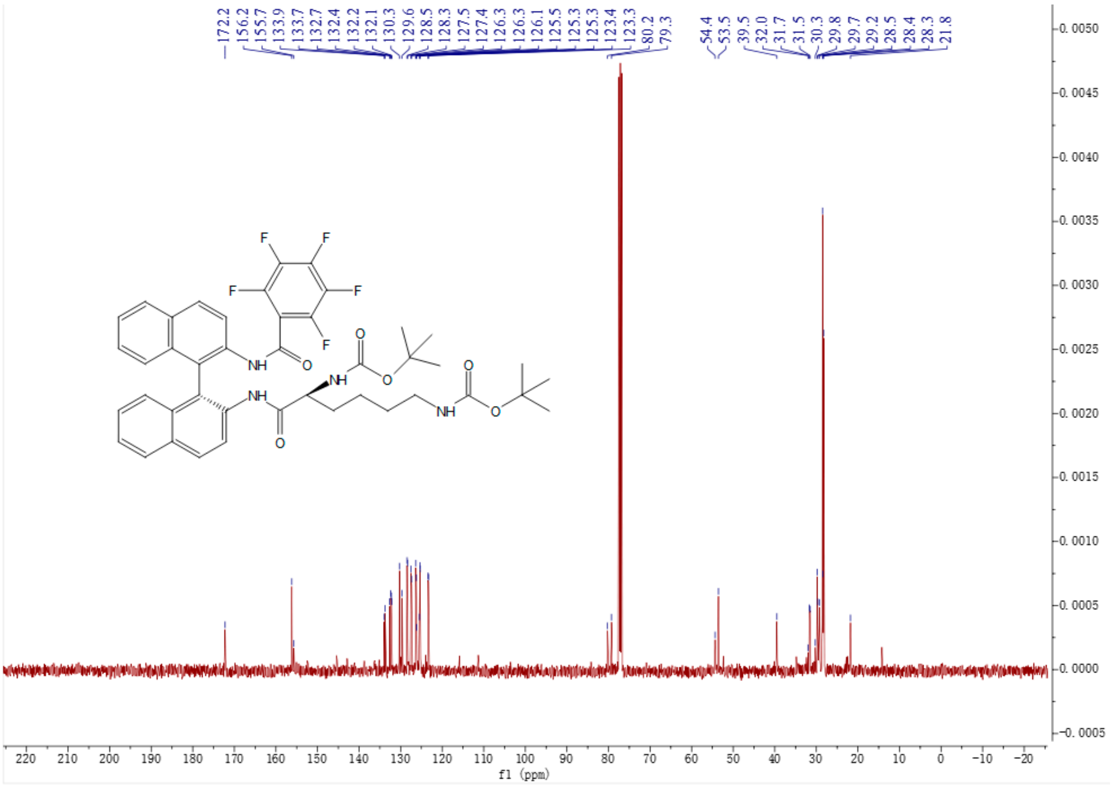
172.2
156.2
155.7
133.9
133.7
132.7
132.4
132.2
132.1
130.3
129.6
128.5
128.3
127.5
127.4
126.3
126.3
126.1
125.5
125.3
125.3
123.4
123.3
80.2
79.3
54.4
53.5
39.5
32.0
31.7
31.5
30.3
29.8
29.7
29.2
28.5
28.4
28.3
21.8
F
NH
O
f1 (ppm)

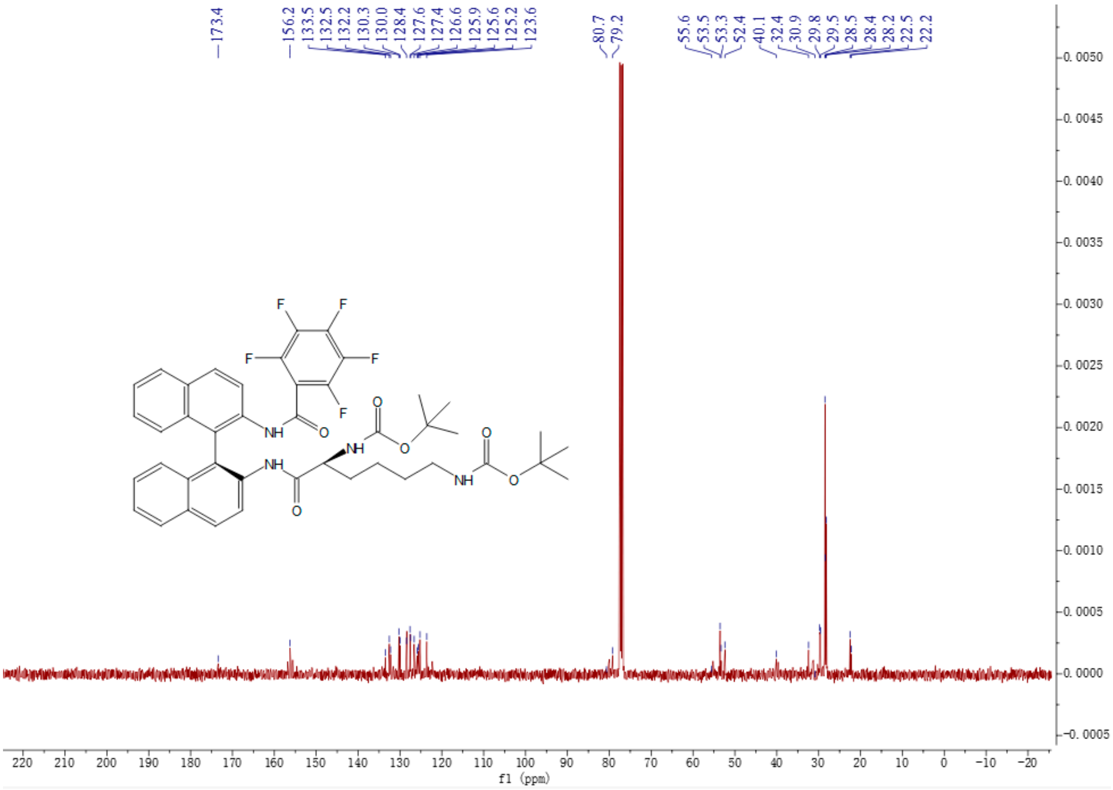
173.4
156.2
133.5
132.5
132.2
130.3
130.0
128.4
127.6
127.4
126.6
125.9
125.6
125.2
123.6
80.7
79.2
55.6
53.5
53.3
52.4
40.1
32.4
30.9
29.8
29.5
28.5
28.4
28.2
22.5
22.2
F
NH
O
f1 (ppm)

**Figure S27:** $^{19}$F NMR spectra of (D/L，R/S)-1.（400M DMSO）

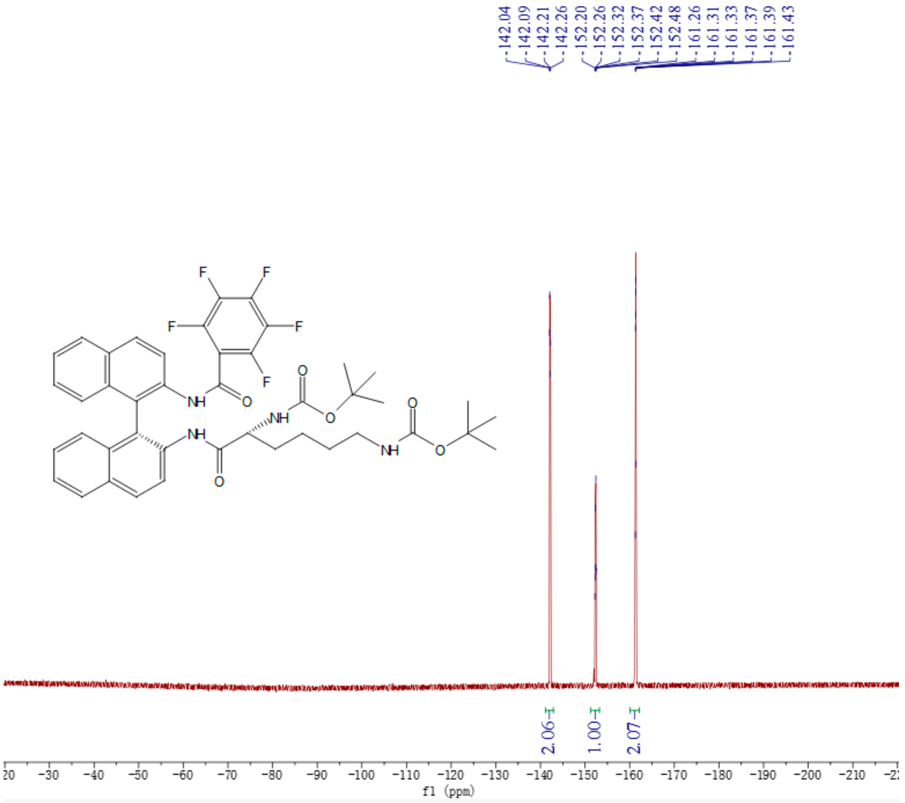

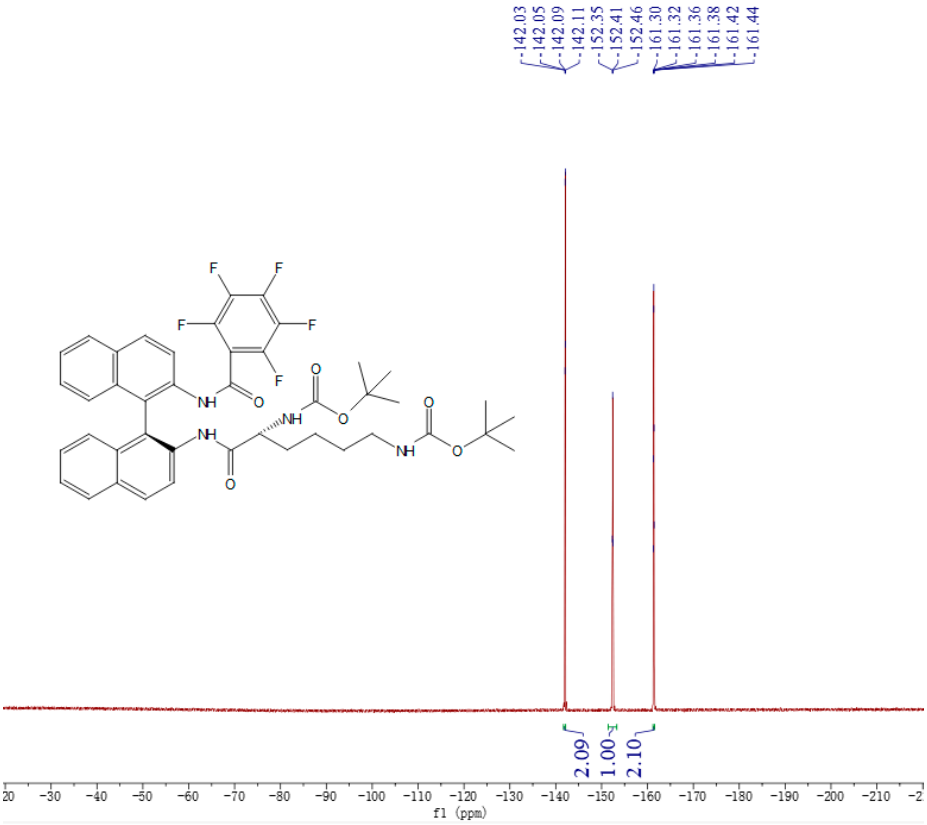

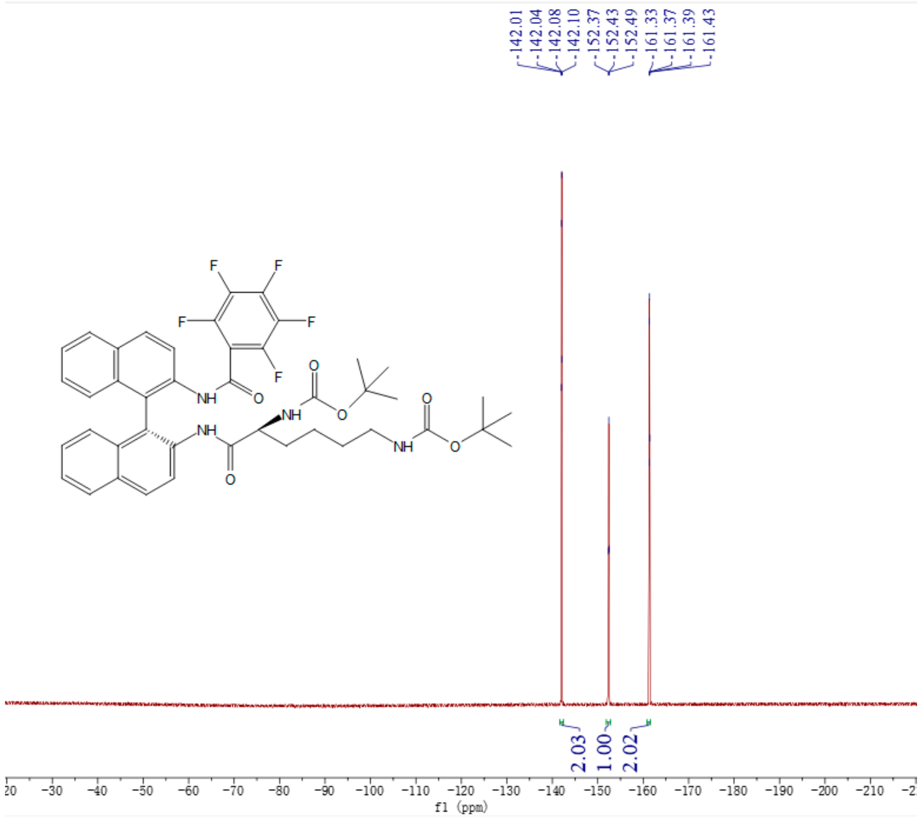
-142.01
-142.04
-142.08
-142.10
-152.37
-152.43
-152.49
-161.33
-161.37
-161.39
-161.43
F
F
F
F
F
NH
O
O
NH
O
O
NH
O
NH
O
2.03
1.00
2.02
-20 -30 -40 -50 -60 -70 -80 -90 -100 -110 -120 -130 -140 -150 -160 -170 -180 -190 -200 -210
f1 (ppm)

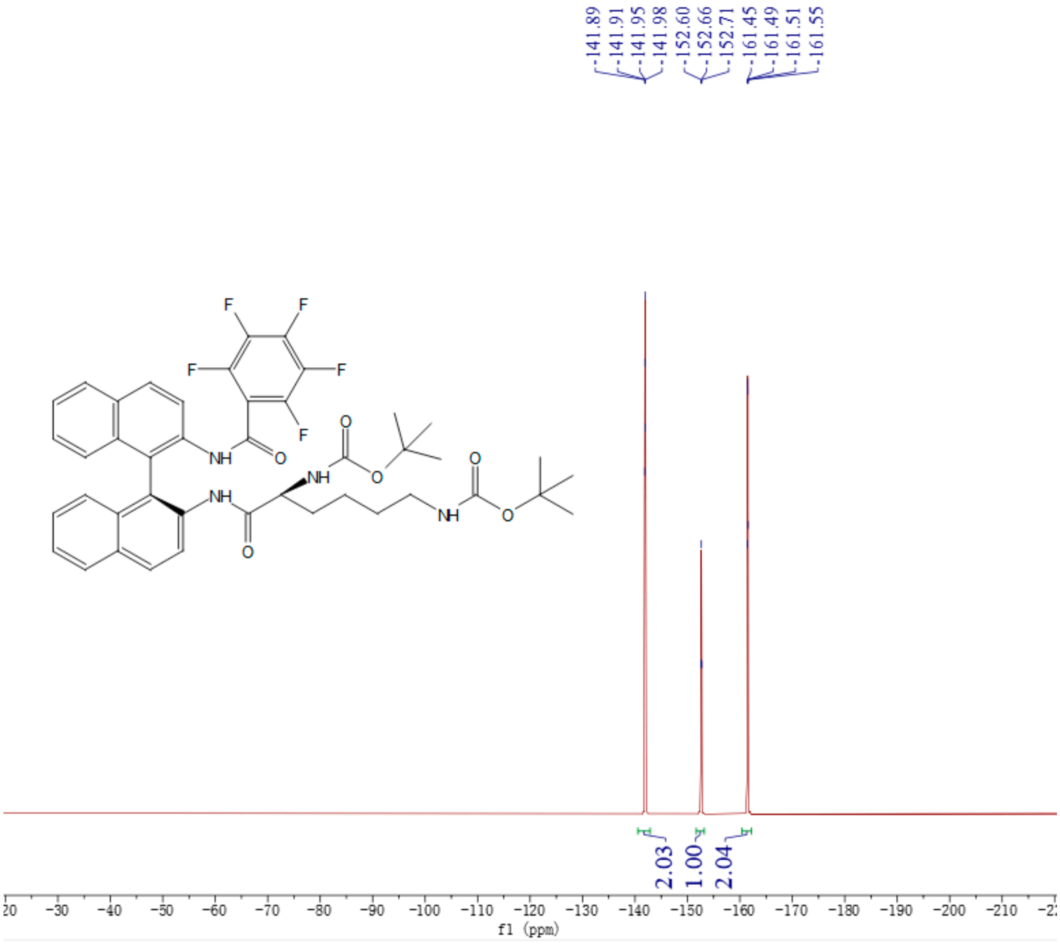
-141.89
-141.91
-141.95
-141.98
-152.60
-152.66
-152.71
-161.45
-161.49
-161.51
-161.55
F
F
F
F
F
NH
O
O
NH
O
O
NH
O
NH
O
2.03
1.00
2.04
-20 -30 -40 -50 -60 -70 -80 -90 -100 -110 -120 -130 -140 -150 -160 -170 -180 -190 -200 -210
f1 (ppm)

**Figure S28:** MS spectra of (D/L，R/S)-1.

| | Formula | Neutral mass | Observed m/z | Mass error (mDa) | Mass error (ppm) | Response | Adducts | Identification status |
|---|---|---|---|---|---|---|---|---|
| (D，R)-1 | C43H43F5N4O6 | 806.31028 | 829.30126 | 1.8 | 2.1 | 5409572 | +Na | Identified |

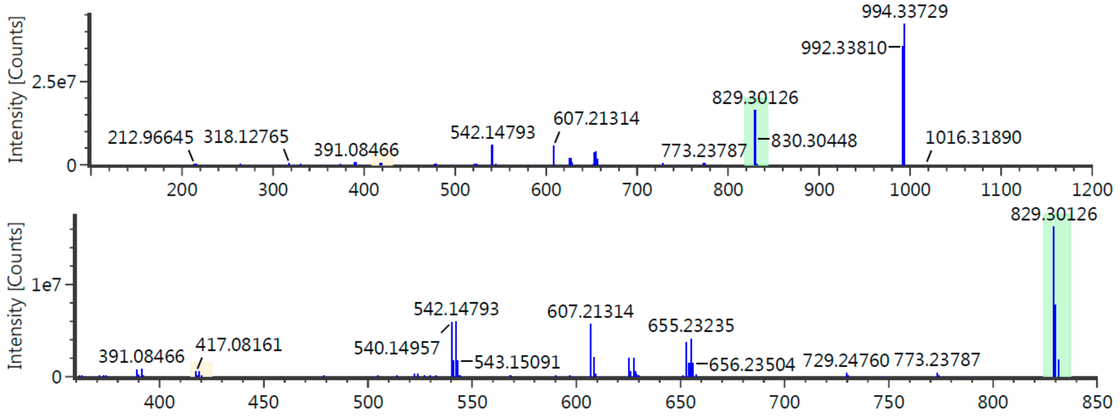


| | Formula | Neutral mass | Observed m/z | Mass error (mDa) | Mass error (ppm) | Response | Adducts | Identification status |
|---|---|---|---|---|---|---|---|---|
| (D，S)-1 | C43H43F5N4O6 | 806.31028 | 829.30017 | 0.7 | 0.8 | 14739752 | +Na | Identified |

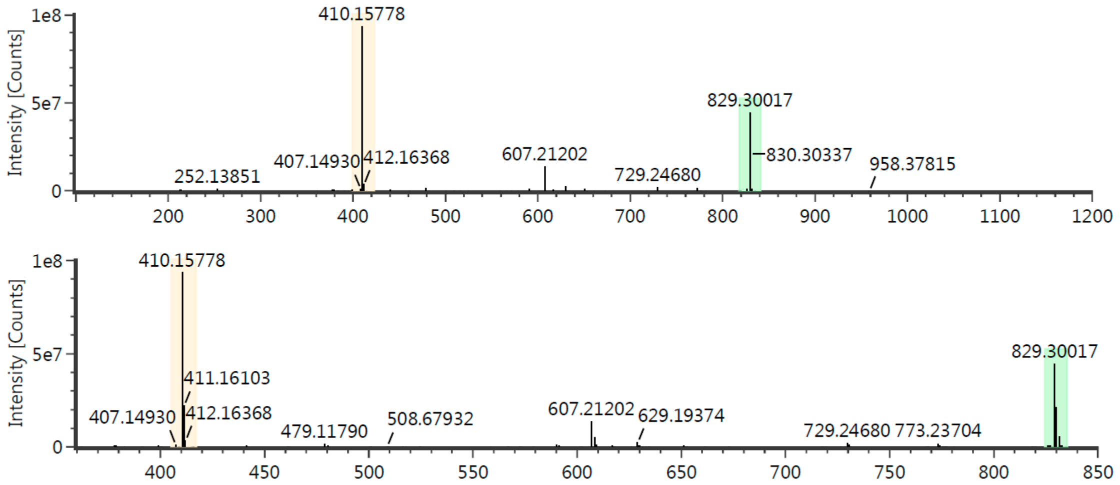

| | Formula | Neutral mass | Observed m/z | Mass error (mDa) | Mass error (ppm) | Response | Adducts | Identification status |
|---|---|---|---|---|---|---|---|---|
| (L，R)-1 | C43H43F5N4O6 | 806.31028 | 829.30097 | 1.5 | 1.8 | 14596839 | +Na | Identified |

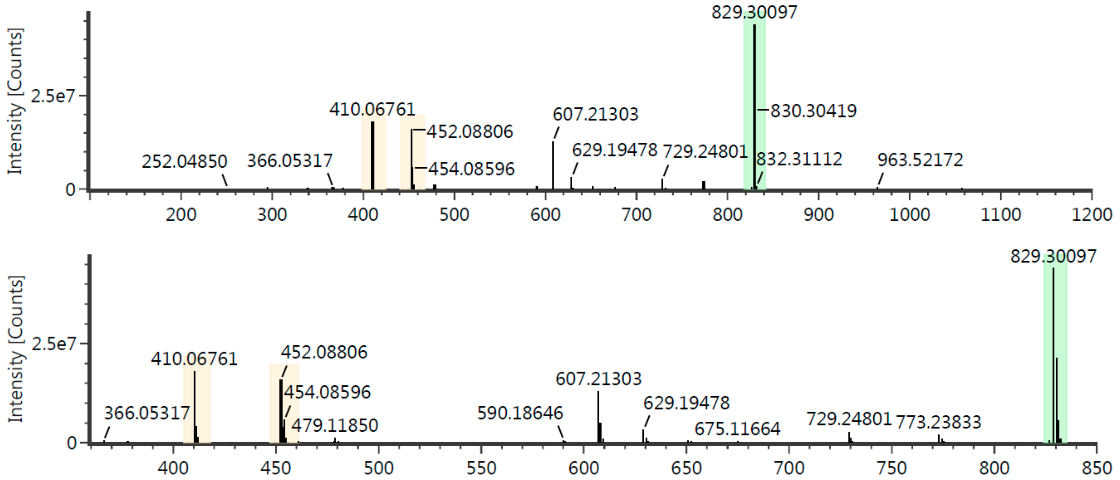


| | Formula | Neutral mass | Observed m/z | Mass error (mDa) | Mass error (ppm) | Response | Adducts | Identification status |
|---|---|---|---|---|---|---|---|---|
| (L，S)-1 | C43H43F5N4O6 | 806.31028 | 829.30089 | 1.4 | 1.7 | 2450287 | +Na | Identified |

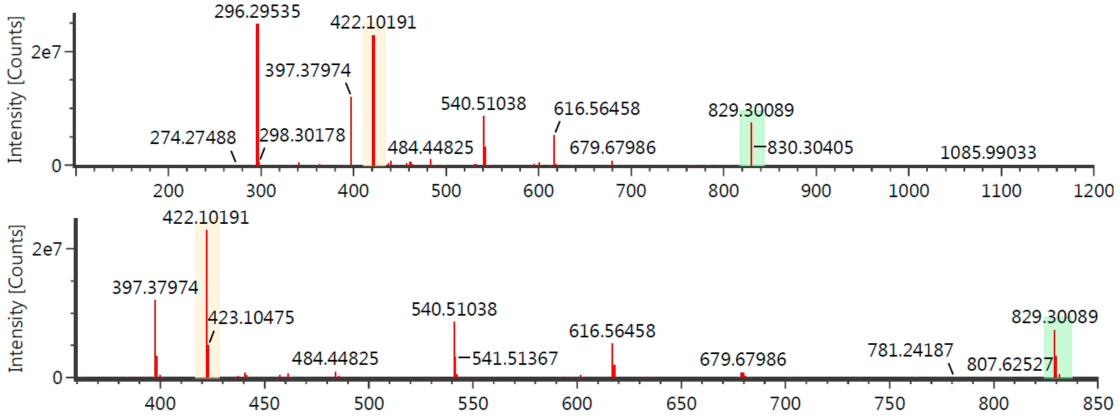


| MS Instrument Type: Waters Vion® IMS QTof | |
|---|---|
| Experiment Settings: | |
| Experiment type: ESI+ | Scan Mode: MS |
| Capillary voltage: 3.0kv | Low mass: 50m/z |
| Soure temperature: 120°C | High mass: 2000m/z |
| Desolvation temperature: 450°C | Scan time: 0.200s |
| Cone gas: 50L/h | Desolvation gas: 800L/h |
